# Web Use for Symptom Appraisal of Physical Health Conditions: A Systematic Review

**DOI:** 10.2196/jmir.6755

**Published:** 2017-06-13

**Authors:** Julia Mueller, Caroline Jay, Simon Harper, Alan Davies, Julio Vega, Chris Todd

**Affiliations:** ^1^ School of Health Sciences University of Manchester Manchester United Kingdom; ^2^ Manchester Academic Health Science Centre Manchester United Kingdom; ^3^ School of Computer Science University of Manchester Manchester United Kingdom

**Keywords:** Online health information, health information seeking, Internet, symptom appraisal, Web search, search strategies

## Abstract

**Background:**

The Web has become an important information source for appraising symptoms. We need to understand the role it currently plays in help seeking and symptom evaluation to leverage its potential to support health care delivery.

**Objective:**

The aim was to systematically review the literature currently available on Web use for symptom appraisal.

**Methods:**

We searched PubMed, EMBASE, PsycINFO, ACM Digital Library, SCOPUS, and Web of Science for any empirical studies that addressed the use of the Web by lay people to evaluate symptoms for physical conditions. Articles were excluded if they did not meet minimum quality criteria. Study findings were synthesized using a thematic approach.

**Results:**

A total of 32 studies were included. Study designs included cross-sectional surveys, qualitative studies, experimental studies, and studies involving website/search engine usage data. Approximately 35% of adults engage in Web use for symptom appraisal, but this proportion varies between 23% and 75% depending on sociodemographic and disease-related factors. Most searches were symptom-based rather than condition-based. Users viewed only the top search results and interacted more with results that mentioned serious conditions. Web use for symptom appraisal appears to impact on the decision to present to health services, communication with health professionals, and anxiety.

**Conclusions:**

Web use for symptom appraisal has the potential to influence the timing of help seeking for symptoms and the communication between patients and health care professionals during consultations. However, studies lack suitable comparison groups as well as follow-up of participants over time to determine whether Web use results in health care utilization and diagnosis. Future research should involve longitudinal follow-up so that we can weigh the benefits of Web use for symptom appraisal (eg, reductions in delays to diagnosis) against the disadvantages (eg, unnecessary anxiety and health care use) and relate these to health care costs.

## Introduction

The Web has become an important resource for lay information about health, with almost three-quarters of the population in developed countries accessing the Web to research health topics [1,2]. The Web is accessed to obtain information on general health topics, such as weight management, and by patients to obtain information on their diagnosed condition [3,4]. Moreover, the Web is accessed to assess and evaluate symptoms and their causes [1].

The way the Web is used by patients who have obtained a specific diagnosis from a health care professional is likely to differ from the way it is used in the absence of a professional diagnosis when appraising symptoms. Postdiagnosis, individuals have specific medical terms they can use as search terms. Most focus their Web search on treatment options, illness management, and prognosis [3,4]. When appraising symptoms with the aim of diagnosing them, on the other hand, most individuals have only symptoms and lay medical knowledge to guide their search, and symptoms are sometimes vague and difficult to describe.

Web use for symptom appraisal may have important implications, although it is unclear whether it plays a beneficial or detrimental role in health care delivery. For example, some evidence suggests it could lead to unnecessary anxiety about health and increase use of health service resources [5]. Other findings imply it could enhance patient empowerment and help patients prepare for consultations [6]. Thus, Web use for symptom appraisal may lead to either wasting or more efficient use of resources. For example, Web use for symptom appraisal may cause anxiety about health by making individuals falsely believe they have a serious condition when they do not. On the other hand, it may encourage people with warning signs to present to health services, promoting earlier diagnosis. It could also falsely reassure people that symptoms are not serious, thus preventing earlier diagnosis. This is particularly important for potentially life-threatening or debilitating conditions, which are easier to treat when detected early, such as cancer [7], heart disease [8], or glaucoma [9]. Understanding Web use for symptom appraisal is also relevant for less serious conditions, such as a common cold, because it could lead people with mild symptoms to present to health services when this is not necessary or it could help people identify the symptoms that can be treated at home. As these examples highlight, Web use for symptom appraisal may have important implications for health care utilization.

To leverage the potential for reducing strain on health care resources and promoting earlier diagnosis, we need to understand the current role of the Web in help seeking and symptom evaluation, and the strategies people use to access information, taking differing contexts into account. Because these questions cannot be addressed in a single study, a systematic review is required, involving a thorough and comprehensive search of the literature, critical appraisal of individual studies, and extraction and synthesis of relevant findings.

This systematic review addresses the following five review questions:

1. What proportion of different populations (eg, general, specific disease, or demographic groups) use the Web to appraise symptoms?
2. Which symptoms are likely to be researched online?
3. How is Web use for symptom appraisal conducted (search strategies)?
4. What are the behavioral consequences of Web use for symptom appraisal?
5. What are the emotional consequences of Web use for symptom appraisal?

## Methods

A protocol was developed by the research team based on the review questions and an initial broad search of the available literature, using the Centre for Reviews and Dissemination’s guidance for undertaking reviews in health care [10] and the Preferred Reporting Items for Systematic Reviews and Meta-Analyses (PRISMA) statement [11].

### Eligibility Criteria

#### Study Focus

We included studies that addressed use of the Web to appraise symptoms (ie, to research symptoms and their potential causes). This could include both actual symptoms and symptoms in fictional scenarios. This did not have to be the primary focus of the study; some reference to Web use for symptom appraisal was sufficient. If studies examined health-related Web use in general, they were screened during full-text review and excluded if no specific reference to symptom appraisal was made. Studies that analyzed anonymous logs were included if they examined symptom-related searches. We included only studies that focused on human behavior; studies that evaluated the performance of Web-based tools were excluded.

#### Populations

Studies on Web use for symptom appraisal of any physical health conditions were included. Studies examining mental health/psychiatric conditions were excluded to focus the scope of the review. Studies on Web use by health professionals were excluded. Studies from any country were included, as long as the publication was written in English.

#### Study Design

Our initial scoping suggested a scarcity of research in this area, thus we did not limit included studies to any particular design. Nonempirical studies (eg, theoretical papers and literature reviews) were excluded.

#### Publication Types

Full paper, English-language publications were included, regardless of the original language of the research.

### Information Sources

We searched PubMed, EMBASE, PsycINFO, ACM Digital Library, SCOPUS, and Web of Science for relevant publications up to September 30, 2016. To minimize publication bias, grey literature was explored by searching OpenGrey, an open-access database containing more than 700,000 bibliographical references of grey literature. We also searched the British Library Integrated catalog, which contains reports, conference abstracts, and theses. Finally, authors in the field were contacted to inquire about any unpublished material, if two or more of their papers were among the included studies, or if their paper was judged as particularly relevant to the review (eg, if examining Web use for symptom appraisal was the primary focus of the study).

### Search

The terms Internet, Web, online, search engine, Google, help seeking, health information seeking, symptom, and diagnosis were entered into the databases (note Google was used as a search term because this is by far the most widely used search engine worldwide [12]). An example search strategy is provided in Multimedia Appendix 1.

### Study Selection

The study selection process followed the guidelines provided in the PRISMA statement [11]. Search terms were entered into the databases and all returned studies were imported into a single Mendeley file. Three independent reviewers assessed the studies for eligibility. Studies were first screened by titles and abstracts. Selected studies were then screened for inclusion by reading full texts. Reference lists of included studies were handsearched for further eligible studies. We also handsearched journals if they contained two or more articles included in this review or if the general journal topic area was particularly relevant to the review, to ensure inclusion of studies not yet loaded on electronic databases. Any discrepancies between the reviewers were discussed until consensus was reached.

### Data Collection Process and Data Items

From each study, any information regarding use of the Web for symptom appraisal was extracted, as well as details on study design, procedure, population, sampling method, entry and inclusion criteria for study participants, sample size, measures, and details of analysis methods (data extraction sheet in Multimedia Appendix 2).

### Quality Appraisal

A quality appraisal of selected articles was conducted based on five criteria designed for reviews incorporating mixed study designs [13] (Multimedia Appendix 3). Quality appraisal involved two stages. First, articles were assessed for inclusion in the review using a relatively liberal threshold; articles were scored eligible if they addressed each criterion at least minimally. Criteria were then applied more rigorously using a three-point scoring system (low/medium/high; see Multimedia Appendix 3) and main limitations of each study were identified. This assessment was used to critically appraise studies during synthesis of the findings.

### Synthesis of Results

The extracted data were synthesized using Thematic Analysis, which has been identified as one of the main approaches used to review and synthesize qualitative and quantitative evidence [14,15]. Our analysis involved the following steps [16]:

1. Data familiarization: familiarization with the data was achieved by reading all included studies several times and extracting the relevant information into the data extraction sheets.
2. A priori grouping: data from the data extraction sheets were grouped according to the review question they pertained to and summarized in a matrix. Studies were entered into the rows of the matrix, whereas study characteristics, limitations, and review questions were entered into the columns. This matrix enabled us to compare the findings of different studies pertaining to the same review question, taking methodological aspects into account (example matrix shown in Multimedia Appendix 4). This method was adapted from Framework Analysis, which is a specific form of Thematic Analysis [17].
3. Generation of initial codes: the data were initially coded using semantic codes within the NVivo10 environment, using the matrix to compare results across studies.
4. Searching for themes: once all data extracts were coded, codes were sorted into broader, more conceptual categories to create themes.
5. Reviewing themes: finally, we reviewed the data extracts the themes related to, to determine whether the created themes satisfactorily captured the raw data.

For quantitative studies reporting proportions without confidence intervals, 95% confidence intervals were computed using the asymptotic (Wald) method based on a normal approximation [18] to facilitate comparisons between studies.

## Results

### Study Selection

**Figure 1:**
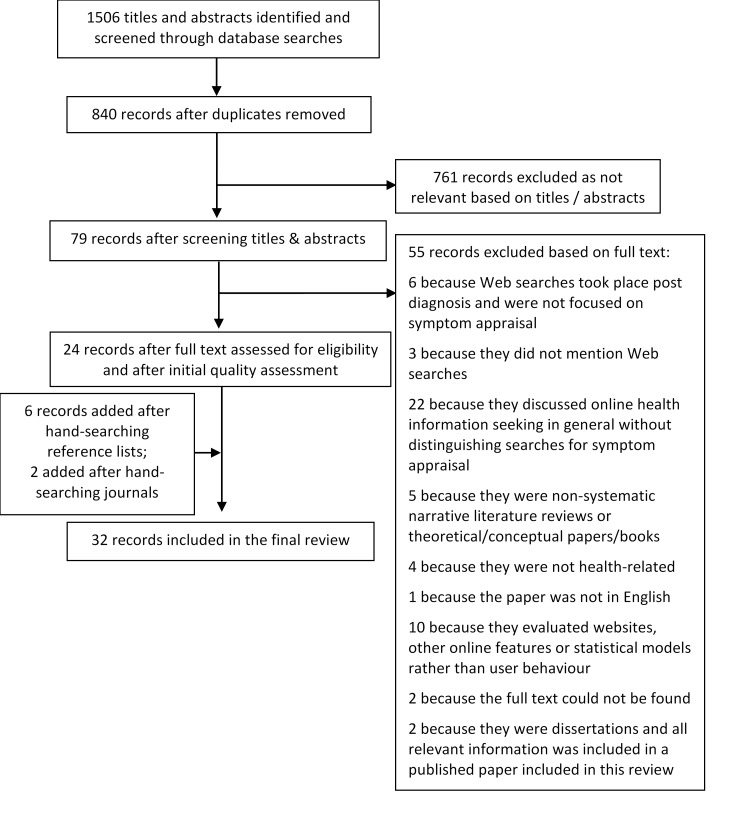
PRISMA diagram for the study identification process.

Thirty-two studies were identified as eligible for inclusion in the review. The search process is illustrated in Figure 1. The grey literature search yielded no further inclusions. The *Journal of Medical Internet Research*, the *Journal of Health Communication*, *Telemedicine and e-Health*, and the *Journal of the American Medical Informatics Association* were handsearched, resulting in 15 full-text assessments and two further inclusions. Four authors were contacted to enquire about unpublished material. We received one reply, concerning an article we had already identified.

### Study Characteristics

Tables 1-3 provide an overview of study characteristics. Study designs included cross-sectional surveys (n=10, two of these with embedded qualitative interview studies, one with an embedded observational study), qualitative interview and focus group studies (n=4), experimental studies (n=7), two studies evaluating questions posed by users to a health website (n=2), a study evaluating clicks on a specific health website (n=1), and studies involving the analysis of log data from search engines (n=8). Two of these combined a log-based approach with a survey. Most studies were conducted in the United States (n=25).

**Table 1:** Study design and aim of the studies included in the review (N=32).

Author, date	Study design	Aim
Attfield et al, 2006 [6]	Qualitative interview study; cross-sectional	Explore information seeking of patients before and after consultations, its situational influences, and its impact on patient-provider relationships
Briet et al, 2014 [19]	Quantitative; cross-sectional analysis of website queries	Explore the nature and content of questions and answers on a health website, and to examine the situations of patients asking questions
Cartrightet et al, 2011 [20]	Longitudinal log-based study	Analyze the search activity of users researching health information online and identify goals and patterns of search behavior
Chin, 2009 [21]	Experimental between subjects design: 2×2 (ill–well-defined tasks, younger-older users)	Compare older and younger adults in their performance and search behavior in ill and well-defined tasks
Chin & Fu, 2010 [22]	Experimental between subjects design: 2×2×2 (older-younger adults, parts-systems interface, parts-system task)	Examine differences between older and younger adults in interacting with different online search tasks and interfaces
Cooper et al, 2013 [23]	Qualitative study (focus groups)	Explore how women would evaluate symptoms associated with gynecologic cancers
Cumming et al, 2010 [24]	Cross-sectional Web-based survey study	Evaluate digital storytelling videos (videos of people talking about their own experiences) about help seeking for menopausal symptoms
De Choudhury et al, 2014 [25]	Cross-sectional survey study (quantitative + qualitative data) + longitudinal log-based study	Research the prevalence of health activities on social media and search engines; characterize health activities on the different platforms and describe how people evaluate information obtained from these
Fiksdal et al, 2014 [26]	Qualitative focus group study	To gain a deeper understanding of online health-searching behavior to inform future developments of personalizing information searching and content delivery.
Fox & Duggan, 2013 [1]	Nationwide cross-sectional survey	The Pew Internet & American Life Project is an initiative of the Pew Research Center, a nonprofit “fact tank” that provides information on the issues, attitudes and trends shaping America and the world
Hay et al, 2008 [27]	Mixed-methods survey and interview study	Understand the extent and reasons for online research prior to first appointments for patients in a rheumatology clinic
Keselman et al, 2008 [28]	Cross-sectional qualitative interview and Think Aloud study.	Explore users’ information-seeking difficulties by conceptualizing information seeking as a form of hypothesis testing, and to examine the role of users’ competencies in online information seeking
Lauckner & Hsieh, 2013 [29]	Experimental 2×2 design (position: top-bottom; frequency: high-low)	Does the position and frequency of serious conditions in search results affect perceived severity and susceptibility, and are they related to negative emotional outcomes? Do health literacy and experience with online health seeking moderate these relationships?
Luger, 2014 [30]	Experimental 2×2 design: two different symptom vignettes (mononucleosis or scarlet fever), either Google or WebMD	Explore older adults’ online health seeking to determine the cognitive and diagnostic processes involved
Medlock et al, 2015 [31]	Cross-sectional online survey	To determine which information resources seniors who use the Internet use and trust for health information, which sources are preferred, and which sources are used by seniors for different information needs
Morgan et al, 2014 [32]	Analysis of inquiries posted to a health website	Describe what information people seek from a US website about genetic and rare diseases, and why
Mueller et al, 2016 [33]	Experimental (randomized trial)	To assess the feasibility of testing a symptom appraisal tool for lung cancer symptoms in an online randomized trial
Norr et al, 2014 [34]	Experimental within-subjects design	Investigate whether viewing medical websites adversely affects anxiety sensitivity
North et al, 2011 [35]	Cross-sectional analysis of clicks on a health website and calls to a telephone triage system	Establish what symptoms Internet users tend to look up online and whether telephone triage algorithms could be applied to these
Perez et al, 2015 [36]	Experimental study with Think Aloud	Describe Internet search processes and identify demographic and personal characteristics associated with use of system 1 (does not include hypothesis testing and evidence gathering) and system 2 (includes hypothesis testing and evidence gathering) processing
Powell et al, 2011 [37]	Cross-sectional survey with embedded qualitative semistructured interviews	Identify the characteristics and motivations of online health information seekers accessing the NHS Direct website
Powley et al, 2016 [38]	Cross-sectional survey and observational study	Evaluate whether patients with inflammatory arthritis and inflammatory arthralgia use the Internet for symptom appraisal and to assess the advice given and diagnoses suggested by the NHS and WebMD symptom checkers
Rice, 2006 [39]	Cross-sectional survey study; secondary analysis of existing dataset	Understand what influences online health seeking, what the reported benefits of online health seeking are, and to identify similarities among online activities
Teriaky et al, 2015 [40]	Cross-sectional survey	Understand how outpatients awaiting initial gastroenterology consultation seek medical information on the Internet and how wait times affect Internet usage
Thomson et al, 2012 [41]	Cross-sectional survey study	Explore characteristics of colorectal cancer patients who used the Web to appraise symptoms prior to diagnosis
White & Horvitz, 2009 [5]	Longitudinal log-based study and cross-sectional survey	(1) Describe escalations that occur when users search for common symptoms and how this escalates to queries about serious conditions, and (2) examine how this persists over several sessions
White & Horvitz, 2009 [42]	Cross-sectional survey study	Explore how lay individuals use the Web to find explanations for symptoms, what activities they pursue, and what their experiences are
White & Horvitz, 2010 [43]	Longitudinal log study using logs from Windows Live toolbar	Predict escalations in searches based on characteristics of websites visited
White & Horvitz, 2010 [44]	Longitudinal log-based study	Establish predictors of when searches for common symptoms lead to health care utilization
White & Horvitz, 2012 [45]	Longitudinal log-based study	Explore how users search for medical concerns and particularly how these concerns impact on future behavior (eg how this influences focus and attention of future searches)
White & Horvitz, 2013 [46]	Longitudinal log-based study	(1) Whether snippets in search results are biased toward serious conditions when symptoms are entered into search engines and 2) how these snippets influence user behavior
Ybarra & Suman, 2006 [47]	National, longitudinal telephone survey	Examine which factors predict whether a Web user is likely to contact a health professional

**Table 2:** Characteristics of the study populations of studies included in the review (N=32).

Author	Study population	Setting	Sample size
Attfield et al [6]	2 groups of 8 NHS patients: 1 group from a Patient Advice and Liaison Service (PALS) patient panel (43-81 years, mean 64) and one group of MSc students for HCI (25-42 years, mean 31)	UK	16
Briet et al [19]	Users asking hand surgery-related questions from a free online health consultation website	USA (American website; no restriction regarding location of website users)	131 questions
Cartright et al [20]	A set of filtered logs from a toolbar deployed by the Windows Live search engine, containing at least 1 symptom	USA (English-language logs, but no restriction regarding location of users)	2,329,231 actions (=queries issued to a search engine)
Chin [21]	Younger and older adults from a university community	USA	69; 41 younger adults (18-35), 28 older adults (60-83)
Chin & Fu [22]	Younger and older adults from community of a medium-sized city	USA	46, 23 younger (18-28) and 23 older (60-77) adults
Cooper et al [23]	Women aged 40-60 years	USA	132
Cumming et al [24]	Visitors of a UK-based menopause website	UK (UK website; no restriction regarding location of website users)	539
De Choudhury et al [25]	Survey: US adults 18-70 years (census representative sampling); Twitter: 15-month sample of Twitter’s Firehose stream, English-language Tweets relating to health; log: data from a major Web search engine	USA (survey with US residents, only English-language log data but not restricted to a certain country)	210 survey respondents; 125,166,549 tweets; 174,605,024 searches
Fiksdal et al [26]	Adult, English-speaking members of the Olmsted County, MN community (where Mayo Clinic is located) and Mayo Clinic patients, employees, and family visitors	USA	19
Fox & Duggan [1]	Adults living in the United States	USA	3014
Hay et al [27]	English-speaking US adults (≥17 years)	USA	120
Keselman et al [28]	Lay individuals (convenience sample)	USA	20
Lauckner & Hsieh [29]	Students from an undergraduate communication course at a large Midwestern university	USA	274
Luger [30]	Older US adults, ≥50 years, community resident, without cognitive impairment, who owned a computer	USA	79
Medlock et al [31]	Members of a local senior (Christian) organization	Netherlands	118
Morgan et al [32]	Random sample of English-language inquiries posted by lay people to the question and answer section of the GARD website and inquiries sent via email	USA (American website but no restrictions on locale of users)	278 inquiries, 68 from 2006 and 210 from 2011
Mueller et al [33]	Adults living in UK with undiagnosed symptoms potentially related to lung cancer	UK	97
Norr et al [34]	Undergraduate students from a large university in the Southern United States.	USA	56
North et al [35]	All symptom assessment callers to Ask Mayo Clinic (telephone triage) and all clicks to specific symptoms on the symptom-checker page of MayoClinic.com	USA	70,370 calls; 2,059,299 clicks
Perez et al [36]	Young adults aged 21-35 with experience of online health information and reported barriers to accessing health services	USA	78
Powell et al [37]	Users of the NHS Direct website	UK	792 for survey, 26 for interviews
Powley et al [38]	Newly presenting patients with either clinically apparent synovitis or a new onset of symptoms consistent with inflammatory arthritis but without clinically apparent synovial swelling attending a secondary care based rheumatology clinic	UK	34
Rice [39]	US adults: respondents from studies conducted within the Pew Internet and American Life project	USA	13,978 respondents in 2000 who reported health seeking online, 500 of these were telephone interviewed in 2001
Teriaky et al [40]	Patients awaiting appointments at a general gastroenterology clinic in London, ON, Canada	Canada	87
Thomson et al [41]	Newly diagnosed colorectal cancer patients (<6 months)	USA	242
White & Horvitz [5]	Log data related to symptom queries (no mention of restriction by locale) from all major Web search engines (eg, Google, Yahoo!, or Live Survey): randomly selected employees of the Microsoft Corporation who had performed at least 1 health-related online search; survey: Microsoft employees	USA (survey with US residents, no restriction mentioned regarding locale for logs)	Logs: 8732 users with symptom-related queries; survey: 515 participants
White & Horvitz [42]	5000 Microsoft employees were invited via email, from these volunteers were chosen who indicated in a prescreening that they searched the Web for medical information	USA	515 survey respondents
White & Horvitz [43]	Logs from windows live browser toolbar, English-speaking USA relating to 6 basic symptoms	USA (log data issued from US locale)	“Many thousands of logs were mined”
White & Horvitz [44]	Logs from consenting Windows live toolbar users over a 6-month period relating to 3 symptoms: chest pain, muscle twitches, abdominal pain	USA (log data issued from US locale)	700 queries with symptom to HUI transition; 700 queries with symptoms to no HUI transition
White & Horvitz [45]	Logs from consenting Windows live toolbar users over a 3-month period	USA (log data issued from US locale)	169,513 queries
White & Horvitz [46]	Log data related to symptoms queries generated in English-speaking US locale	USA (log data issued from US locale)	2070 symptom queries from 714 users
Ybarra & Suman [47]	Americans living throughout the 50 states and the District of Columbia	USA	Year 1=2104; year 4: 2010, 570 of these were year 1 participants

**Table 3:** Nature of measures and procedures of studies included in the review (N=32).

Author	Nature of measures and procedure
Attfield et al [6]	Semistructured interviews, eliciting accounts of health information-seeking episodes and how they relate to ongoing health care
Briet et al [19]	Questions and answers to a health website were categorized and analyzed descriptively
Cartright et al [20]	Logs were mined and categorized as either evidence-directed, hypothesis-directed with diagnostic intent, or hypothesis-directed with informational intent, according to defined algorithms
Chin [21]	Participants were randomized to complete either an ill-defined task (find possible causes for a list of symptoms) or well-defined task (find a specific medical term), using a health website; cognitive measures (working memory capacity, processing speed), health literacy measures, medical knowledge measure, search performance for both tasks were measured
Chin & Fu [22]	Participants were given a symptom vignette and asked to find possible causes. Participants were randomized to complete either a parts task (described symptoms based on body parts) or a systems task (described symptoms by functional systems). Tasks were completed either in the parts interface (categorized symptoms based on body parts) or systems interface (categorized symptoms based on functional body systems). Measures included Patients’ Medical Background Knowledge, Mental Interface Match Index, Broadness (no. of links), link decision time: time spent reading.
Cooper et al [23]	Discussion in focus groups: which symptoms from a list would be of most concern, why, and what could cause them, what would be their hypothetical response to them, what were actual responses in the past?
Cumming et al [24]	Participants viewed a storytelling video online and then completed a questionnaire evaluating the effect of the video on feeling informed, planned future help seeking, etc
De Choudhury et al [25]	In the survey, participants were asked questions about their experiences using Twitter and search engines to share and seek health information; on the log analysis, tweets and logs were categorized as relating to 4 categories: (1) symptoms of major diseases, (2) benign explanations (nonlife-threatening illnesses), (3) serious illnesses, and (4) disabilities; logs were then analyzed descriptively
Fiksdal et al [26]	Moderators used a semistructured moderator guide to facilitate discussion in focus groups about: (1) participants’ perception and understanding of health care information, (2) the process of information collection on the Internet, (3) understanding and usage of information, and (4) implications of health care information for health and well-being
Fox & Duggan [1]	People were contacted via telephone for telephone interviews about online health information seeking
Hay et al [27]	Before their appointment, patients were interviewed about online health information (OHI) seeking, and completed the Wong-Baker-Faces Pain Scale; the consultation was audio-recorded to determine whether OHI was mentioned and then patients completed a satisfaction scale regarding the consultation
Keselman et al [28]	Participants read a hypothetical scenario describing a relative who experienced symptoms typical of stable angina and then discussed possible causes of symptoms from the symptom vignettes in semistructured interviews; then Think Aloud while they researched symptoms on MedlinePlus
Lauckner & Hsieh [29]	The study took place online; participants were presented with a symptom vignette and then with a search engine result page manipulated to show serious conditions either at the top or bottom, and low or high frequency of serious conditions; participants then completed several scales: perceptions of severity and susceptibility using the Risk Behavior Diagnosis scale, history of viewing online health information, their health status, how often they experienced each of the 4 symptoms, and their demographic information, health literacy using the Newest Vital Sign (NVS)
Luger [30]	Participants were presented with 1 of 2 symptom vignettes and asked to diagnose them using Think Aloud, either on Google or WebMD. Measures taken included Think Aloud, self-reported age, gender, ethnicity, education, and income, recent health history, number of hours per week that they used a home computer as well as the number of years that they had owned a home computer, whether or not they had previous experience with the Internet tool to which they were assigned (Google or WebMD’s Symptom Checker).
Medlock et al [31]	Participants completed an online questionnaire, which included questions about health information resources used; the Autonomy Preference Index was used to assess information needs and preferences for involvement in health decisions
Morgan et al [32]	A random sample of questions posted to the GARD website were analyzed thematically; collected data included inquiry origin (domestic), type of contact (email and Web-based form), gender, date received at the information center, the specific condition for which they were inquiring, primary language (English), and their reason for inquiry
Mueller et al [33]	Participants first completed a survey about their symptoms and risk factors. They were then randomized to receive the intervention (personalized, theory-based health webpages), or control conditions. Subsequently, participants completed a questionnaire which assessed demographic details, participants’ self-reported intention to seek help (scale 1-7), behavioral attitudes and beliefs about help seeking.
Norr et al [34]	Participants first completed the Anxiety Sensitivity Index (ASI), Intolerance of Uncertainty Scale (IUS), and a health anxiety scale (SHAI). Participants were randomized to view either symptom-related websites or general health and wellness control websites. Afterwards, they completed the ASI and SHAI.
North et al [35]	For the MayoClinic website, click data was collected using Google Analytics; for the telephone triage, all completed calls were counted and put into symptom categories based on the algorithm/guideline used during the call.
Perez et al [36]	Participants were randomized to one of two symptom scenarios and instructed to search the Internet while using Think Aloud; participants’ Internet searches and think-out-loud vocalizations were digitally recorded using screen capture video-recording software
Powell et al [37]	Users of the NHS Direct website completed an online questionnaire survey. A subsample of survey respondents participated in in-depth, semistructured, qualitative interviews by telephone or instant messaging/email.
Powley et al [38]	Patients completed a brief survey on Internet use for symptom appraisal prior to attending clinic; patients were then asked to complete the NHS and WebMD symptom checkers based on their symptoms and their answers and the outcomes were recorded; demographic and disease-related data were obtained from clinic records.
Rice [39]	Respondents were contacted via telephone for telephone interviews asking about online health seeking.
Teriaky et al [40]	Patients awaiting gastroenterology consultation were asked to complete a questionnaire consisting of 16 multiple-choice questions to understand patient use of Web resources for medical information. Abstracted information included patient demographics, level of education, reason for referral, preceding investigations, patient resources utilized, websites browsed, information obtained, reasons for seeking information on the Internet, patient self-diagnosis, and lifestyle changes instituted.
Thomson et al [41]	Semistructured interviews focused on patient sociodemographic and psychological factors, symptom recognition and appraisal, and communication with HCPs, friends, and family.
White & Horvitz [5]	Analysis of logs: Formulated a list of symptoms and associated benign and serious conditions. Recorded all queries to search engines and clicks on result pages, and identified those that included symptoms as search terms. Escalations: Observed increases in medical severity of search terms within a search session. Nonescalations: Search progresses to benign explanation of the symptom; survey: Microsoft employees were sent a survey with open and closed-ended questions regarding participants’ medical history and online search behavior
White & Horvitz [42]	Microsoft employees were sent a survey to elicit perceptions of online medical information, experiences in searching for this information, and the influence of the Web on health care concerns and interests. The survey contained “around 70” open and closed questions
White & Horvitz [43]	Cases were identified where queries for symptoms were followed by a query about a related serious condition. Cases where it led to a benign query or no change were termed nonescalations. Using logistic regression, a model was developed to predict escalation using website features of the previously visited page; website features: structural features, title and URL features, firs-person testimonials, page reliability/credibility, commercial intent
White & Horvitz [44]	Log analysis: logs containing symptoms as search terms were filtered, and it was determined whether subsequent searches showed health care utilization intent (HUI). Logistic regression was used to predict HUI based on search characteristics; log entries include a user identifier, a timestamp for each page view, and the URL of the page visited; HUI: queries that indicate searching for contact information for medical facilities
White & Horvitz [45]	Queries were labeled to identify medical and symptoms related queries, and escalations. Subsequently occurring searches were examined. Log entries included a unique user identifier, a timestamp for each page view. Search sessions on Google, Yahoo!, and Bing. Escalation queries were categorized as within-session and between session
White & Horvitz [46]	Log data relating to symptom queries were filtered. Subsequent behavior on the search engine result page was examined, including hovering, cursor movements, clicks, scrolling, as well as bounding boxes of *areas of interest* (AOIs)
Ybarra & Suman [47]	Respondents were contacted via telephone and completed a telephone survey about online health information seeking and help-seeking behavior (seeking help from a health professional or others)

As Table 4 shows, some studies explored Web use regarding current symptoms (n=5) or symptoms that had been experienced previously (n=7), or both (n=1), whereas other studies examined Web use for symptom appraisal by providing participants with a symptom vignette and instructing them to imagine they have these symptoms (n=8). In several studies (n=11), the exact situation of participants was unclear because anonymous data were collected online. Table 4 also highlights the variety and nonspecificity of symptoms examined; most studies (n=15) examined general symptoms and although 10 studies examined specific conditions, only two studies examined similar conditions [27,38]. Finally, most studies (n=20) did not follow up whether participants had received a diagnosis.

**Table 4:** Symptoms and diagnoses examined in included studies.

Author, date	Were participants symptomatic, asymptomatic, or previously symptomatic^a^?	Type of symptoms examined	Did the study follow up whether Web use was followed by a diagnosis?
Attfield et al [6]	Previously symptomatic	General (any symptoms)	Not assessed
Briet et al [19]	Unclear, participants were users asking questions about symptoms^b^	Hand illness-related symptoms	Not assessed
Cartright et al [20]	Unclear, participants were users issuing symptom-related queries to a search engine^b^	General^c^	Not assessed
Chin [21]	Asymptomatic, participants were presented with a symptom vignette	Symptom vignettes included: pain and stiffness in the body; burning, itching, and sometimes tingling sensation on their body; feeling feverish and chilly after an overseas trip; fatigue, sudden weight gain and difficulty dealing with cold; however, results were not analyzed separately for different symptoms	Not applicable^d^
Chin & Fu [22]	Asymptomatic; participants were presented with a symptom vignette	General (participants received 6 different vignettes with different symptoms, not assessed separately)	Not applicable^d^
Cooper et al [23]	Asymptomatic; participants were presented with a list of symptoms	Symptoms related to gynecologic cancers	Not applicable^d^
Cumming et al [24]	Most symptomatic (448/492), but some asymptomatic (44/492)	Menopausal symptoms	Not assessed
De Choudhury et al [25]	Unclear, participants were users issuing symptom-related Tweets and queries to a search engine^b^	General, logs were filtered for references to symptoms using a comprehensive list of symptoms from the Merck medical dictionary	Not assessed
Fiksdal et al [26]	Previously symptomatic	General (any symptoms)	Not assessed
Fox & Duggan [1]	Previously symptomatic	General (any symptoms)	Participants were asked whether their diagnosis was confirmed by a health professional; 45% said it was confirmed, 35% did not present, 19% said it was not confirmed/inconclusive
Hay et al [27]	Symptomatic; participants were newly diagnosed rheumatology patient	Rheumatoid symptoms	Yes, patients’ diagnoses were gathered after the appointment or at follow-up appointment
Keselman et al [28]	Asymptomatic; participants received a symptom vignette	Symptoms typical of stable angina	Not applicable^d^
Lauckner & Hsieh [29]	Asymptomatic; participants received a symptom vignette	Symptom vignettes involved one of four symptoms: headaches, chest pain, muscle twitches, or abdominal pain, but the different symptoms were not analyzed separately	Not applicable^d^
Luger [30]	Asymptomatic; participants received a symptom vignette	Symptom vignettes involved either mononucleosis or scarlet fever	Not applicable^d^
Medlock et al [31]	Previously symptomatic	General (any symptoms)	Not assessed
Morgan et al [32]	Unclear, participants were users issuing symptom-related Tweets and queries to a search engine^b^	Symptoms related to any type of genetic or rare disease	Not assessed
Mueller et al [33]	87 participants were symptomatic, 10 were asymptomatic but searching on behalf of someone else	Symptoms related to lung cancer	Not assessed
Norr et al [34]	Asymptomatic; participants viewed a list of symptoms	General (“websites focused on symptoms of medical conditions”)	Not applicable^d^
North et al [35]	Unclear, participants were users searching the MayoClinic website or using a telephone triage^b^	General (any symptoms)	Not assessed
Perez et al [36]	Asymptomatic; participants received a symptom vignette	One of two clinical symptom scenarios: (1) fever, mild headache, dry cough, and myalgia, suggestive of influenza, and (2) fever, severe headache, and stiff neck, suggestive of meningitis	Not applicable^d^
Powell et al [37]	Unclear, participants were users of the NHS website^b^	General (any symptoms)	Not assessed
Powley et al [38]	Symptomatic; participants were patients attending a secondary care based rheumatology clinic	Either clinically apparent synovitis or a new onset of symptoms consistent with inflammatory arthritis but without clinically apparent synovial swelling	Yes, rheumatological diagnosis was recorded after consultation
Rice [39]	Previously symptomatic	General (any symptoms)	Not assessed
Teriaky et al [40]	Symptomatic; participants were patients awaiting gastroenterology appointments	Symptoms related to gastroenterology	Not assessed
Thomson et al [41]	Symptomatic; participants were colorectal cancer patients	Symptoms related to colorectal cancer	Yes; all participants were diagnosed with colorectal cancer
White & Horvitz [5]	Logs: Unclear, participants were users issuing symptom-related queries to a search engine^b^; survey: previously symptomatic	Logs related to 3 common symptoms (headache, muscle twitches, and chest pain)	Not assessed
White & Horvitz [42]	Previously symptomatic	General (any symptoms)	Not assessed
White & Horvitz [43]	Unclear, participants were users issuing symptom-related queries to a search engine^b^	Queries related to any of 6 common symptoms: headache, chest pain, muscle twitches, abdominal pain, nausea, and dizziness	Not assessed
White & Horvitz [44]	Unclear, participants were users issuing symptom-related queries to a search engine^b^	Queries related to one of 3 symptoms: chest pain, muscle twitches, and abdominal pain	Not assessed
White & Horvitz [45]	Unclear, participants were users issuing symptom-related queries to a search engine	General^c^	Not assessed
White & Horvitz [46]	Unclear, participants were users issuing symptom-related queries to a search engine^b^	General^c^	Not assessed
Ybarra & Suman [47]	Previously symptomatic	General (any symptoms)	Not assessed

^a^ Symptomatic: participants experienced the symptoms at the time of the study; asymptomatic: participants did not have symptoms and were surveyed regarding fictional symptoms; previously symptomatic: participants were surveyed about symptoms they experienced previously.

^b^ Participants were users asking questions about symptoms (could be own symptoms or asking on behalf of someone else).

^c^ Any queries related to a comprehensive list of symptoms from the Merck medical dictionary.

^d^ Patients were not symptomatic.

### Quality Assessment and Risk of Bias

Quality assessment of the studies is shown in Multimedia Appendix 3. Subsequently, we use this information to critically appraise evidence from the studies and assess risk of bias.

### What Proportion of Different Populations Use the Web to Appraise Symptoms?

Four studies, all surveys, reported the proportion of the study sample that engaged in Web use for symptom appraisal (Table 5).

**Table 5:** Percentage of people engaging in Web use for symptom appraisal reported by included studies (n=4).

Reference	Study population	Sample size	Reported Web use for symptom appraisal, % (95% CI)
Fox & Duggan [1]	Adults living in the US	3014	35% (33%-37%)
White & Horvitz [42]	US Microsoft employees	515	75% (71%-79%)
Medlock et al [31]	Members of a senior church organization, Netherlands	118	23% (15%-31%)
Thomson et al [41]	Colorectal cancer patients, US	242	25% (20%-31%)

In Fox and Duggan’s [1] population-based survey with adults living in the United States, 35% reported going online to attempt self-diagnosis. Participants were sampled to mirror the population in terms of demographics, but disproportionately stratified to increase the incidence of nonwhite respondents. This survey was conducted in the United States and is therefore likely to reflect proportions in Western, high-income countries with high Internet penetration.

White and Horvitz’s [42] survey conducted among Microsoft employees found that “three-quarters of subjects” (the authors do not provide absolute numbers; assuming the proportion is 75% of N=515, 95% CI 71%-79%) reported searching for information on symptoms. “Two-thirds” reported researching professionally undiagnosed conditions, at least once a month [42]. It should be noted that this sample was biased toward younger, male respondents with high educational level and socioeconomic status working within an industry that is very Web-oriented.

Medlock et al [31] examined online health information seeking in older people by surveying members of a senior Christian organization. They found that 23% of participants reported using the Web in the past 12 months to determine the cause of symptoms. This shows that, although Web use for symptom appraisal may be less common among older people than in the general population (compared to 33%-37% found by Fox and Duggan [1]), older people do engage in it.

While the previous surveys focused on diagnostic searches for any conditions/symptoms, Thomson et al [41] conducted a survey with colorectal cancer patients and found that 25% of the sample reported prediagnosis Web use for symptom appraisal.

To conclude, Fox and Duggan’s [1] study with its large, population-based sample size is most likely to give an accurate proportion for the general population (in a Western, higher income country), although the other included studies give an indication of how this proportion can vary depending on the population being surveyed (ie, depending on sociodemographic variables and disease-related factors). It should also be noted that the confidence intervals are wide in a number of these studies reflecting considerable uncertainty about the true proportion.

### Which Symptoms Are Likely to Be Researched Online?

Six studies examined characteristics of symptoms that were searched for online [25-27,35,39,41]. Three of these were survey studies [27,39,41], one was an interview study [26], and two involved analyses of data on usage of online resources such as social media, search engines, and health websites [25,35].

North et al [35] compared users of the Mayo Clinic website with people who used a telephone triage system to appraise their symptoms and found that telephone triage users were more likely to have acute and conspicuous symptoms requiring immediate relief, whereas website users were more likely to research chronic conditions. Hay et al [27] surveyed rheumatology patients and found that some individuals in their study sought help online because they had a history of undiagnosed symptoms. Findings from both of these studies suggest symptoms are researched online when they have been present for a prolonged time.

In their study on colorectal cancer patients, Thomson et al [41] found that neither symptom severity nor stage at diagnosis was related to Web use for symptom appraisal, but Web users were more likely to experience symptoms typically perceived as embarrassing, such as change in bowel habits. Similarly, Choudhury et al [25], who analyzed log data obtained from Twitter and a search engine, found that potentially embarrassing, stigmatized, or sensitive symptoms such as “vaginal bleeding” or “pelvic pain” were more likely to be searched for than tweeted. Furthermore, Rice’s [39] population-based telephone survey conducted in the United States concluded that more frequent online health seekers were more likely to look for sensitive health topics that are difficult to talk about than less frequent online health seekers.

Finally, in Fiksdal et al’s [26] focus group study with 19 US adults, participants reported turning to the Web when symptoms were perceived as trivial/nonserious and they wanted to avoid “bothering” health professionals.

In conclusion, it appears Web use for symptom appraisal occurs when symptoms are persistent, have a history of being undiagnosed by health professionals, are potentially embarrassing or stigmatized, and/or when they are perceived as superficial/nonserious.

### How Is Web Use for Symptom Appraisal Conducted (Search Strategies)?

#### Theme 1: Symptom-Based, Condition-Based, and Treatment-Based Searches

Three distinct approaches to searching were identified: (1) symptom-based searches, which used symptoms as search terms; (2) condition-based searches, which involved searches for particular conditions, and (3) treatment-based searches, which involved researching treatments for symptoms without prior research on possible causes.

Log data from search engines suggest the majority (65%) of exploratory health-related searches (ie, those aimed at diagnosing a condition) are symptom-based rather than condition-based [20], and remain symptom-based throughout the search because search sessions tend to start and end with purely symptom-related queries [5,20]. One should bear in mind, however, that log-based studies cannot ascertain searchers’ actual intentions and motivations. The authors assume occurrences of certain search terms signal certain intentions (eg, a symptom and the term “cause” signals diagnostic intent); however, articles did not report any prior validation of these algorithms.

An experimental study that observed people (N=79) as they used Google or a symptom-checker tool to diagnose symptom vignettes reported that most users conduct symptom-based searches because most people began their search by entering symptoms and only 24% began by specifying a condition [30].

In an experimental study conducted by Perez et al [36], participants (N=78) were instructed to research the Web as if they were experiencing a given symptom, described in a vignette, while using Think Aloud. Think Aloud (also known as “cognitive interviewing”) requires participants to vocalize their thoughts while performing a task [48]. The authors found that 19% of searches were treatment-based and the remainder symptom- or condition-based (the authors did not report these separately). It should be noted that the external validity of vignette-based studies is limited because individuals base their searches on the vignette descriptions rather than actual perceptions or observations, and the search behavior observed is likely to depend on the phrasing of the vignette.

Keselman et al [28] used interview and Think Aloud methods to explore how a convenience sample of 20 lay individuals interpreted a symptom vignette using the American consumer health information service MedlinePlus. They concluded that some participants conducted condition-based searches and some participants used a symptom-based approach. Additionally, they identified a group of participants who used a condition-based approach, but began their search with a broader hypothesis, such as “heart disease,” and then attempted to narrow down their search. Different barriers seemed to play a role in the different search strategies: condition-based searchers were prone to confirmation bias, seeking out information that confirmed their hypothesis and terminating the search before reviewing further hypotheses. Those starting with a broad hypothesis often terminated the search without coming to a conclusion. The symptom-based searchers struggled to find the relevant results due to the lack of specificity of their search terms [28].

Overall, it seems most Web use for symptom appraisal searches are symptom-based and both log-based studies, which have high external validity, and experimental studies, which have high internal validity, confirm this finding. No validation was reported for the algorithms used for the log-based studies, however, and experimental and qualitative studies used to observe search behavior have limited generalizability to real-world contexts.

#### Theme 2: Selection of Search Terms

Keselman et al [28] examined the search behavior of 20 adults using Think Aloud and discovered that participants often ignored symptoms mentioned in the vignette if they perceived them as irrelevant and exempted these from their search terms. The authors termed this “selective perception bias.” The participants also tended to ignore aspects of duration of the symptoms and had difficulty discerning acute from chronic symptoms. However, these findings stem from a single study with only 20 adults using fictional scenarios, thus further validation is required.

#### Theme 3: Age Differences

Three studies reported on age differences in search behavior [21,22,30]. Chin et al [21] (N=69) compared the search performance of younger and older adults while performing either a well-defined task (searching for a specific medical term on a website) or an ill-defined task (using the website to diagnose a set of symptoms). The study found that older adults performed better in the ill-defined task, whereas younger participants performed better in the well-defined task [21].

In another study (N=46), Chin and Fu [22] presented older and younger adults with different interfaces of the same website: one interface categorized symptoms based on the body parts they occurred in and the other interface categorized symptoms according to functional systems (eg, respiratory system). Younger adults tended to click on significantly more links within one category, suggesting they followed the interface of the website, whereas older adults clicked significantly more between-category links regardless of the interface. Chin and Fu [22] conclude this was due to older adults using their existing medical knowledge rather than the interface to guide their search, which is supported by their finding that older adults performed better in a medical knowledge task [22].

Luger at al [30] explored the search behavior of adults aged 50 years and older (N=79) in a Think Aloud study and found that participants who accurately diagnosed the condition presented in a symptom vignette were slightly younger (mean 61.72, SD 6.17 years) than those who were inaccurate (mean 65.51, SD 7.54 years), although no inferential statistics were reported.

Thus, there are some indications that older adults perform differently in Web searches for symptom appraisal than younger adults, possibly due to medical knowledge. However, the available studies used small sample sizes, thus inferences to the wider population may not be appropriate.

#### Theme 4: Selecting Information

Several studies examined how users select information from their search results. We identified four subthemes relating to selection of information.

##### Number of Search Results Viewed

Lauckner and Hsieh [29] reported that participants in their laboratory-based, experimental study with undergraduate students (N=274) viewed approximately four links on results pages, which was the number of results visible above the “fold” (ie, users would need to scroll down to view more results). Corroborating this finding, Keselman et al [28] discovered that participants in their qualitative study (N=20) often ignored relevant links while trying to diagnose a symptom vignette if these were located below the fold. Luger et al [30] found that older adults (N=79) in their experimental, laboratory-based study tended to view approximately six conditions on the WebMD symptom-checker tool after entering a set of symptoms, although the authors do not clarify whether this was the number visible above the fold.

Thus, the top results returned by search engines will have maximum impact on symptom appraisal, whereas those located below the fold may have little to no effect. Because these findings all relate to laboratory-based studies, however, further investigation in naturalistic settings would be beneficial.

##### Process of Elimination

In their study using Think Aloud with 79 adults aged 50 years and older, Luger et al [30] found that 91% of participants used a “process of elimination,” whereby the symptoms described in the vignette were compared against those listed for a given condition and the condition was discarded as a hypothesis if it included symptoms not mentioned in the vignette. This finding suggests that a common search strategy is to narrow down the hypotheses by discarding those with symptoms not matching one’s own. However, the majority of the sample was highly educated (all had some university education), therefore generalizability to the wider population is unclear.

##### Source Credibility

In Luger et al’s [30] study with adults aged 50 years and older (N=79), source credibility was mentioned by only 25% of the sample. They also found that one-third viewed user-generated content such as discussion boards, which are not quality controlled. White and Horvitz [46] found in their log-based study that consumer sites such as MayoClinic.com or WebMD (both well-known American corporations and health websites) are positively related to click-through rates following searches that contain symptoms as search terms, suggesting widely known, established health websites are likely to be accessed during Web use for symptom appraisal. White and Horvitz [5] also suggest searches that “escalate” (ie, progress from searching for symptoms to serious conditions) contain more visits to “trusted sources” (eg, governmental websites, websites of health organizations). It is important to note that although the authors claim to research “diagnostic searches,” this was identified through the presence of symptoms in search terms and may therefore also include nondiagnostic searches.

##### Pages Mentioning Serious Illnesses

White and Horvitz [46] filtered logs from a search engine and examined how users issuing symptom queries subsequently interacted with search results pages. They found that users engage more with captions on search results pages that mention serious illnesses, hovering more frequently and longer over these captions and clicking these more often than captions mentioning benign causes. Terminology related to serious illnesses such as “malignant,” “severe,” and “tumor” significantly increased click probability, whereas terms such as “benign” decreased click probability. Additionally, users were more likely to engage with sites indicating they can help identify causes of symptoms (eg, by mentioning the words “learn” and “causes”).

Although we do not know searchers’ intentions or how they used the information found, these findings suggest those researching symptoms online are more likely to engage with websites relating to serious causes.

To summarize, Web use for symptom appraisal typically involves inputting information into a search tool and subsequently narrowing down results returned by the search tool. When inputting information, most users appear to choose search terms based on symptoms rather than hypothesized conditions, but users do not appear to utilize all information available (eg, some symptoms may be omitted, as well as the frequency/duration of symptoms). Furthermore, there is some limited evidence that older adults perform differently in Web searches for symptom appraisal than younger adults, and that this may be due to medical knowledge. Once a selection of results is provided by the search tool, users tend to narrow results down by taking into account the results’ position on the results page, the degree of seriousness of the condition, the credibility of the source, and the extent of overlap between the listed and the experienced symptoms.

### Behavioral Consequences of Web Use for Symptom Appraisal

#### Theme 1: Increased Help Seeking

In Fox and Duggan’s [1] population-based survey (N=3014), 46% (95% CI 44.22%-47.78%) of online self-diagnosers claimed that information found online led them to think they needed the attention of a health professional. Thomson et al [41] found in their survey with colorectal cancer patients that 25% (95% CI 19.54%-30.46%) of online self-diagnosers were reportedly persuaded by the information found online to see a health professional. This suggests the proportion of people encouraged to seek medical help based on Web use for symptom appraisal may be significantly lower among colorectal cancer patients than in the general population surveyed by Fox and Duggan [1].

Using logistic regression with a survey sample of more than 2000 Americans aged 12 years and older, Ybarra et al [47] found that online self-diagnosers were 2.5 times more likely to report contacting a health professional than online health information seekers who did not try to diagnose symptoms online, suggesting Web use for symptom appraisal is linked to increased health care contact.

Some studies suggest that the mode of presenting information on a website may affect users’ decisions to seek medical advice: in a UK-based qualitative study [24], participants reportedly felt encouraged to seek help after viewing an online “digital storytelling” video about urogenital atrophy; 73% who had reportedly been too embarrassed to see a health professional before and 87% who had not wanted to bother their doctor would now seek help. In an online pilot randomized trial, Mueller et al [33] examined whether addition of theory-based components to online health information can increase intention to seek help. The theory-based components appeared to significantly increase intention to seek medical help, although the sample size in this pilot study (N=97) was too small to allow firm conclusions.

Using log-based search engine data, White and Horvitz [44] examined search behaviors related to health care utilization intent. Health care utilization intent was assumed to be present when users conducted searches for health care practitioners/clinics near their geographical area. They found that users who displayed certain search behavior—such as visiting websites that mention serious conditions before benign ones—were more likely to show health care utilization intent subsequently. This suggests online search behavior following symptom queries is related to subsequent health care contact, although mechanisms of causality are unclear based on this data. Furthermore, the authors do not report whether/how their algorithms were validated; thus, it is unclear whether their proxy measure of health care utilization intent is valid.

By observing how patients attending a rheumatology clinic completed the NHS and WebMD symptom-checker tools, Powley et al [38] found indications that symptom-checker tools provide information that can propagate unnecessary help seeking. Of 34 patients, 15 were inappropriately advised to seek help from emergency services rather than primary care, indicating potential issues with the algorithms used in symptom-checker tools. One should bear in mind, however, that this study does not allow any conclusion on whether real users would follow this advice or not.

#### Theme 2: Decreased Help Seeking

In Powell et al’s [37] interview study of users (N=26) of the NHS Direct website (the official website of the UK National Health Service), some participants reportedly used online health information as a form of “demand management,” to identify trivial symptoms not warranting medical attention. Similarly, some participants in Fiksdal et al’s [26] focus group study reported using the Web to avoid “bothering” health professionals with trivial symptoms. In both studies, it was not followed up whether users had correctly or incorrectly classified symptoms as trivial. Finally, in Attfield et al’s [6] interview study, participants reported sometimes being reassured by Web searches that help seeking was not necessary. Evidence for this theme stems only from qualitative studies, thus, generalizability is uncertain.

#### Theme 3: Communication with Health Professionals

Fox and Duggan [1] found that 53% of online self-diagnosers reportedly discussed the health information found online with a health professional. Two qualitative studies by Cooper et al [23] and Attfield et al [6] found that patients used the Web to appraise symptoms in order to prepare for consultations by preparing questions, collating relevant information, and enhancing their knowledge in order to understand the advice received.

Two studies found indications that Web use for symptom appraisal is related to reduced communication with a health professional [27,41]. Hay et al [27] found that new rheumatology patients who engaged in Web use for symptom appraisal were significantly less likely to want to challenge their health professionals’ advice than those who did not. The authors note that study participants were concerned about evoking the impression of questioning health professionals’ advice. Thomson et al [41] found that Web use for symptom appraisal was significantly related to feeling hesitant about discussing symptoms with a health professional. However, direction of causality is unclear. It is possible that information found online dissuaded individuals from communicating with health professionals or that people chose to research symptoms online because they were reticent about discussing their symptoms.

From the preceding findings, we can conclude that Web use for symptom appraisal is used to inform the decision of whether to present to health services and that online self-diagnosers are more likely than other health information seekers to contact a health professional. This can potentially be increased, where appropriate, with novel methods such as “digital storytelling” or theory-based components. Some evidence also suggests that online health information can potentially reduce help seeking by calming users’ fears. It is unclear, however, what proportion of users feel encouraged or discouraged to seek help appropriately (ie, what proportion of users who feel encouraged to seek help actually have a condition warranting medical attention, and what proportion of users who feel discouraged to seek help actually do not need medical attention). Furthermore, it is unclear whether those engaging in Web use for symptom appraisal are more or less likely to seek medical advice than those experiencing the same symptoms without researching online because this comparison was not made in any of the included studies. Web use for symptom appraisal can also play a role in communication with health professionals by influencing how individuals prepare for consultations and prompting discussion of online health information.

### Emotional Consequences of Web Use for Symptom Appraisal

In White and Horvitz’s [42] survey among 515 Microsoft employees, 38.5% reported that online health information had made them feel anxious in the past, and 50.3% reported Web use for symptom appraisal had made them feel less anxious. The survey sample was biased toward younger, more educated, and information technology-literate respondents.

Powell et al [37], who examined the motivations of users of the NHS Direct website using semistructured interviews, found that participants sought health information online to obtain reassurance about symptoms. The majority nevertheless subsequently sought medical help, although sometimes with less urgency and anxiety.

Teriaky et al [40] surveyed patients (N=87) awaiting appointments at a general gastroenterology clinic and asked those who reported using the Web prior to their consultations whether this had changed their anxiety levels. In all, 77% experienced no change, 21% experienced an increase, and 2% a decrease. One should note that this sample consists of those who decided to report to health services (and who admitted Web use for symptom appraisal); there may be a larger proportion of users who felt calmed by their searches and therefore did not present to health services.

Lauckner et al [29] found in their experimental laboratory-based study (N=274) that presenting search engine results relating to serious conditions before benign conditions and a higher frequency of results relating to serious conditions was related to negative emotional outcomes such as fear. These findings suggest a causal relationship between exposure to search results during Web use for symptom appraisal and increases in anxiety.

Another experimental study conducted by Norr et al [34], however, found no difference in the anxiety levels of their participants (N=56) following review of either (1) websites containing information on causes of symptoms or (2) websites on general health and wellness (eg, exercise, healthy diet) without reference to medical conditions or symptoms.

Therefore, some evidence suggests there is a relationship between Web use for symptom appraisal and health anxiety. Findings from experimental studies were mixed regarding causal relationships.Surveys and interviews indicate there is a potential for calming effects and decreases in anxiety, and that the proportion who report feeling calmed by Web use for symptom appraisal is higher than those reporting anxiety. It is also possible that those who engage in Web use for symptom appraisal are more anxious about their health generally. It is unclear when anxiety is warranted because participants’ actual diagnoses were not followed up, and comparisons to those who did not research symptoms was lacking.

## Discussion

This is the first systematic review and synthesis of the literature available on Web use for symptom appraisal. Our main findings were:

1. Approximately 35% of the general population engage in Web use for symptom appraisal, but the proportion can vary considerably (25%-75%) depending on the population under study.
2. Symptoms tend to be researched online when they are long term, potentially embarrassing/stigmatized, have been presented to health services previously with inconclusive outcomes, and/or when they are perceived as trivial.
3. Searches tend to be based on symptoms rather than hypothesized conditions; users seem to focus on particular symptoms while disregarding other symptoms and aspects such as frequency and duration.
4. Once a selection of results is returned by the search tool, people use specific techniques to narrow results down (eg, taking into account the position on the results page or the credibility of the source).
5. Evidence indicates that online information is used to inform the decision of whether to contact health services and is related to (increased and decreased) anxiety, but the precise impact cannot be discerned due to lack of follow-up and appropriate comparison groups.

Subsequently, we discuss whether Web use for symptom appraisal should be viewed as an asset or a liability in health care delivery based on currently available evidence, and make recommendations for the improvement of online health information.

### Web Use for Symptom Appraisal: Assistance or Hindrance to Health Promotion?

Criticisms of online self-diagnoses include concern over unnecessary anxiety and health care utilization [5,49]. Our review confirms that the Web can increase anxiety and health care contact among users [5,29,42,44], but reveals that there is insufficient evidence to conclude this occurs unnecessarily.

First, it is important to note limitations of approaches used to examine relationships between Web use for symptom appraisal and health anxiety or help-seeking behavior. Cross-sectional surveys cannot show direction of causality. It is possible that using the Web to appraise symptoms causes anxiety, or that anxiety triggers Web use for symptom appraisal, or that a third factor influences both. Furthermore, the surveys that reported on anxiety among online self-diagnosers were biased toward certain demographic [42] or patient groups [40], and did not use validated measures of anxiety levels.

Log-based studies, which evaluate behavior based on search engine log data, do not allow firm conclusions regarding users’ actual behaviors and motivations. For example, White and Horvitz [5] found that users who begin their searches for symptoms often progress to researching serious conditions, but it is not clear whether users are anxious or using the information to reassure themselves. The authors assume certain search terms signal certain intentions (eg, a symptom and the term “cause” signals diagnostic intent), but no action was described to determine the validity of these assumptions. Thus, insights from log-based studies are limited.

Experimental research shows that users asked to research certain symptoms may report feeling anxious following Web searches [29], but it is not possible to infer whether this anxiety would be unwarranted in a naturalistic setting (ie, if symptoms were actually present).

Using the Web to appraise symptoms may also decrease anxiety in some cases [37,42] and Web searches are sometimes used to identify alternatives to health care utilization [6,37]. Individuals describe using online information to evaluate mild/superficial symptoms to avoid wasting health professionals’ time [26]. This shows that Web use for symptom appraisal can also decrease anxiety and help seeking. It is possible that Web use for symptom appraisal discourages help seeking for trivial symptoms, thus reducing pressure on health care resources. However, it is also possible that Web use for symptom appraisal leads to complacency and prevents help seeking when it is actually necessary.

There are also indications that Web-based information can help individuals recognize their symptoms as signs of serious conditions [41]. Indeed, our review highlights that online health information is an important resource when obtaining information from health professionals is difficult (eg, when symptoms are embarrassing or stigmatized) [25,39,41] or when previous visits to health care have been ineffective [27,35]. This suggests there is potential for the Web to be an assistance to health care.

Finally, it should be noted that worry can also have positive effects on health behaviors [50]. The Protection Motivation Theory suggests that fear will increase intention to perform a certain behavior if the individual feels able to perform the behavior and believes that the behavior will reduce the threat [51]. Similarly, according to the Health Belief Model, fear should result in recommended health behavior if perceived benefits of the recommended behavior are high and barriers are low [51]. Overall, this indicates anxiety induced through online health information can enhance recommended health behaviors if information is presented in a way that enables concrete action and decision making.

A limitation we discovered across different methodologies was the lack of follow-up on participants’ help-seeking behavior and diagnoses. Without this information, we cannot discern whether individuals’ self-diagnoses and decisions regarding help-seeking behavior are appropriate or not. We also cannot determine long-term impacts on health care utilization. Furthermore, essential comparison groups are generally lacking. For example, it would be necessary to compare those who research symptoms online with those who do not (rather than surveying only online self-diagnosers), and to compare those who present to health services with those who do not (rather than surveying only patients presenting in clinic) to determine impacts of Web use.

### Recommendations to Improve Online Health Information

Based on the findings of this review, we suggest changes to health websites, Web apps, and search engines such that they can provide useful information to those researching symptoms.

Our analyses reveal that users tend to search inductively based on symptoms. Search engines and symptom-checker tools need to ensure users are directed to useful information when symptoms are entered. The review also shows that searchers tend to omit dimensions such as duration and frequency of symptoms in their search terms [28], and that symptom-based searchers struggle to find relevant results due to lack of specificity of their search terms. This suggests it is important that users are directed to useful terms to narrow their search and prompted to provide information on duration and frequency of symptoms to improve specificity of searches. In support of this, recent research suggests that incorporation of query expansion techniques into information retrieval systems can improve the search effectiveness of search engines for diagnostic symptom searches [52].

Our review also reveals that online health information can impact on the decision to seek help and on communication with health professionals. Health websites and apps need to ensure they provide useful information to support searchers in their decisions and health care interactions. Health websites providing symptom information should, for example, provide clear guidelines on when medical advice should be sought (eg, if a symptom has a certain quality or duration) and how help should be sought (eg, immediately via emergency services or within the next week via primary care).

### Strengths and Limitations

As the review includes a diversity of study types and methods, a quantitative synthesis or meta-analysis was not possible. However, traditional forms of systematic review that do not make use of all forms of evidence often do not take differing contexts into account, limiting their use to policy makers and practitioners [15]. More inclusive forms of review that combine findings from different study designs allow a richer, more holistic understanding of the phenomenon under study [15]. We were able to combine real-world insights from observational studies, such as analyses of search engine log data with data from more controlled, experimental settings, thereby improving external and internal validity. Furthermore, by incorporating findings from large, population-based studies as well as smaller interview-based studies, we were able to gain an understanding of the impact of Web use for symptom appraisal at the population level, while also obtaining more detailed reports of peoples’ perceptions and experiences. Moreover, by including studies that cover a broad range of populations (eg, different conditions/symptom profiles, age groups, socioeconomic status), we have shown how Web usage can differ depending on context.

In this review, we considered a diversity of symptoms and conditions; when more research in this area becomes available, it would be useful to carry out more focused reviews because the nature of the symptom is likely to influence Web use online [25-27,35,39,41].

Finally, it should also be noted that this review did not examine Web use for mental health symptoms. Web use for symptoms related to mental health and its impact on help seeking represent an important field of study and should be assessed in a separate review of the literature.

### Conclusions and Future Work

This systematic review indicates that the Web can disseminate information to those worried about symptoms and can affect their decisions to present to health services. It also suggests Web use for symptom appraisal can impact on how patients prepare for consultations with health care professionals. Thus, we can conclude that Web use for symptom appraisal has the potential to influence the timing of help seeking and the communication between patients and health care professionals during consultations.

At present, limitations of the reviewed studies mean it is not clear when the Web plays a beneficial role in health care delivery and when it is detrimental. Web use for symptom appraisal has been linked to increased as well as decreased anxiety and health care contact. However, the evidence does not show when this is warranted because most studies did not follow up whether participants ultimately sought help following their Web searches and whether they received a diagnosis. Furthermore, comparison groups are lacking to determine the effects of Web use for symptom appraisal.

We need longitudinal research that follows up whether participants seek help and are ultimately diagnosed following Web searches, and compare Web searchers to non-Web searchers. These data can then be used to weigh the benefits of Web use for symptom appraisal (eg, reductions in delays to diagnosis and avoidance of unnecessary health care use) against the disadvantages (eg, unnecessary anxiety and health care use) and relate these to health care costs. Research should focus on real-world samples of people experiencing symptoms and could involve novel methods of tracking behavior, such as analysis of search engine log data and mobile geotracking as used in some of the included studies to follow people over time. These studies have the advantage of high external validity and large sample sizes. However, the algorithms used to analyze these data should first be tested extensively for reliability and validity before further work to evaluate cost effectiveness can meaningfully be conducted. Moreover, further experimental studies would allow a detailed analysis of search behavior. Future research could examine how the different search strategies identified here—symptom-based, condition-based, and treatment-based—relate to cognitive biases and link this to theory.

## Appendix

Multimedia Appendix 1 Example search strategy.

Multimedia Appendix 2 Data extraction sheet.

Multimedia Appendix 3 Quality appraisal of included studies.

Multimedia Appendix 4 NVivo Matrix Screenshot.

## Abbreviations

PRISMA: Preferred Reporting Items for Systematic Reviews and Meta-Analyses
