# Supplementary material for: Web Use for Symptom Appraisal of Physical Health Conditions: A Systematic Review
Source: J Med Internet Res. 2017 Jun 13;19(6):e202. doi: 10.2196/jmir.6755 (PMC5487739; doi:10.2196/jmir.6755)
Supplement: Multimedia Appendix 1 [file jmir_v19i6e202_app1.pdf]

Multimedia Appendix 1

Pubmed example search strategy

((web OR internet OR "search engine" OR google OR online OR “on line”) AND ("help seeking" OR "help-seeking" OR "information seeking" OR "information-seeking") AND (symptom OR symptoms OR diagnoses OR diagnosis))
