# Supplementary material for: Web Use for Symptom Appraisal of Physical Health Conditions: A Systematic Review
Source: J Med Internet Res. 2017 Jun 13;19(6):e202. doi: 10.2196/jmir.6755 (PMC5487739; doi:10.2196/jmir.6755)
Supplement: Multimedia Appendix 2 [file jmir_v19i6e202_app2.pdf]

Multimedia Appendix 2

Data extraction sheet

**Reference number**							
**Title:**							
**Author:**							
**Source:**							
**Date:**		**Vol:**		**Part:**		**Pages:**	
**Objective:**							
**Setting:**							
**Study design:**							
**Procedure:**							
**Study population:**							
**Sampling method:**							
**Entry and exclusion criteria for participants:**							
**Sample size:**							
**Measures**							
**Nature of measures:**							
**Quantitative analysis**							

**What statistical tests were used?:**	
**Power calculation?:**	
**Qualitative analysis**	
**Methodology?**	
**Synthesis method?**	
**Relevant findings:**	
**Authors' conclusions:**	
**Limitations**	
**Reviewer's conclusions**	
