# Supplementary material for: Web Use for Symptom Appraisal of Physical Health Conditions: A Systematic Review
Source: J Med Internet Res. 2017 Jun 13;19(6):e202. doi: 10.2196/jmir.6755 (PMC5487739; doi:10.2196/jmir.6755)
Supplement: Multimedia Appendix 3 [file jmir_v19i6e202_app3.pdf]

Multimedia Appendix 3. Quality appraisal of studies included in the systematic review.

Low = Poorly addressed: Reported but only vaguely ; threats to the validity/reliability/objectivity of the results are probable
Medium = Addressed with minor omissions/issues: Reported but more clarification is needed (stated but details are lacking)
High = Fully addressed: Clearly reported, easy to find and understand in the text; appropriate methods to ensure high validity/reliability/objectivity

Note: For studies with mixed methods (e.g. log analyses and survey), the two methods were assessed separately to show the different methodological issues.

**Authors, date**	**Are the aims and objectives of the research clearly stated?**	**Is the research design clearly specified and appropriate for the aims and objectives of the research?**	**Do the researchers provide a clear account of the process by which their findings were reproduced? (methods)**	**Do the researchers display enough data to support their interpretations and conclusions?**	**Is the method of analysis appropriate and adequately explicated?**	**Main limitations**
Attfield, Adams, & Blandford, 2006	High<br>The aim is described succinctly in a clear sentence. The aim was to explore information seeking and use and related situational factors, and its impact on patient-provider relationships.	High<br>It is a qualitative design which is appropriate for the aims of exploring.	Medium<br>Study methods, measures and analysis are described, but more details on their implementation of Grounded Theory would help.	Medium<br>Identified concept are highlighted by participants' quotes, but the authors do not state how many participants commented on each theme.	Medium<br>Grounded Theory is appropriate for the analysis of qualitative data, but for the specific questions posed here a more structured approach might have been better (e.g. Framework Analysis). Some more details on synthesis method could have been supplied.	Retrospective self-reported accounts – may be biased and/or inaccurate. No inferential/causal conclusions possible
Briet et al., 2014	High<br>The aim is described succinctly in a clear sentence. The aim was to explore characteristics of questions asked by users on a free health consultation website.	High<br>It is made clear this is a cross-sectional design, analysing the questions asked on a health website.	High<br>Methods are described clearly. The health website used is described, and inclusion/exclusion criteria for the questions.	High<br>Interpretations and conclusions are based on quantitative and qualitative results.	High<br>Questions were coded according to categories developed by the researchers and stated explicitly. They were then analysed using descriptive quantitative methods. This is a suitable method for characterizing questions posted to the website.	Unclear how specific the questions were – e.g. did they always allow unambiguous inferences about timing of the questions (pre/post help-seeking)?<br>Were there several coders?

**Authors, date**	**Are the aims and objectives of the research clearly stated?**	**Is the research design clearly specified and appropriate for the aims and objectives of the research?**	**Do the researchers provide a clear account of the process by which their findings were reproduced? (methods)**	**Do the researchers display enough data to support their interpretations and conclusions?**	**Is the method of analysis appropriate and adequately explicated?**	**Main limitations**
Cartright , White & Horvitz, 2011	High<br>The aim is described succinctly in a clear sentence. The aim was to analyse the search activity of users researching health information online, and to identify goals and patterns of search behaviour.	Medium<br>The study aims to examine patterns in online health searches, therefore a log-based study analysing logs from a search toolbar is appropriate. However the authors also want to explore intentions – log data without input from users cannot provide definite information on intentions.	Medium<br>The data used for the study is described in detail, as well as criteria for identifying different types of search sessions. But it is not clear whether/how the authors validated their algorithms.	Medium<br>The study is based on over 2 million queries submitted to a search toolbar. The authors analyse this descriptively. However the authors draw inferences about users' intentions from their search terms alone – and search terms were analysed using computer-based algorithms meaning there is a risk of misclassification.<br>No validation of the algorithm	High<br>The authors describe in detail how they identified certain search types from the logs, how they filtered these from the 2 million search queries and which criteria they used to identify certain behaviours in the users.	Log based study – assumes that certain queries relate to certain topics based on certain algorithms. Some queries may therefore be wrongly classified or the algorithms may not assess what they assume to assess.
Chin, 2009	High<br>The aim is described succinctly in 3 clear sentences. The aim was to compare older and younger adults in their performance and search behaviour in ill and well defined tasks. The research questions are clearly listed.	High<br>2x2 within-subjects design was chosen to compare younger and older adults.	High<br>The study procedure and measures used are described in detail. It is described how variables are operationalised and measured.	Medium<br>Conclusions are based on results of ANOVA and path model, but are partly speculative, e.g. speculation that older adults use existing medical knowledge, but this was not assessed (it was only assessed whether they use an interface-driven strategy).	Medium<br>ANOVA and Hierarchical multiple regression to create path model are used. ANOVA is suitable to compare groups across different conditions. The path model is suitable to create a model of search behaviour. Sample size seems small for ANOVA; power calculation is missing.	The sample size (n = 69) seems small for a 2x2 design. The 'older adults' group (n = 28) was considerably smaller than the younger adults group (n = 41). Quasi-experimental: We cannot draw causal conclusions about causal effects of age on search behaviour/performance. Participants are only from University community – biased sample. Experimental setting: limited external validity
Chin & Fu, 2010	High<br>The aim is described succinctly in a clear sentence. The aim was to examine differences between older and younger adults in interacting with different online search tasks and interfaces.	High<br>2x2 within-subjects design. Appropriate to compare groups and interactions between conditions.	High<br>Methods are described. Study procedure and experimental conditions are described.	Medium<br>Conclusions are based on statistical analyses (ANOVA) but are partly speculative, e.g. speculation that older adults use existing medical knowledge, but this was not assessed.	Medium<br>ANOVA was used which is appropriate to compare groups across different conditions and analyse interactions. Sample size seems small for ANOVA; power calculation is missing.	The sample size (n = 46) seems small for a 2x2 design. Quasi-experimental: We cannot draw causal conclusions about causal effects of age on search behaviour/performance. Participants are only from University community – biased sample. Experimental setting: limited external validity

Authors, date	Are the aims and objectives of the research clearly stated?	Is the research design clearly specified and appropriate for the aims and objectives of the research?	Do the researchers provide a clear account of the process by which their findings were reproduced? (methods)	Do the researchers display enough data to support their interpretations and conclusions?	Is the method of analysis appropriate and adequately explicated?	Main limitations
Cooper, Polonec, Steward, & Gelb, 2013	High<br>The aim is described succinctly in a clear sentence. The aim was to explore how women would evaluate symptoms associated with gynaecologic cancers.	High<br>Qualitative study (focus groups). Appropriate for the aim of exploring perceptions/motivations.	High<br>Methods and the sample are described and how focus groups were moderated and which questions were asked of participants. Qualitative analysis including development of codes is described in detail.	High<br>The authors illustrate how interpretations are based on data by including examples of quotes and indicating how many women endorsed which statements.	High<br>Qualitative analysis is appropriate for the exploratory aim and the focus group-based data. Development of codes is in line with the aims of the study. Attempts to reduce subjectivity of coding (e.g. multiple coders) were made.	Retrospective and speculative self-reported accounts (‘what would you do if…’) – may be biased and/or inaccurate.
Cumming et al., 2010	High<br>The aim is described succinctly in a clear sentence. The aim was to evaluate how digital storytelling impacts on help seeking for menopausal symptoms.	Low<br>Cross-sectional web-based survey. This is not appropriate to assess the impacts of the website as it did not include a control group.	Low<br>Methods and procedure are described. More details on the survey and which questions it included would have been helpful (this can be deduced from the results section but should be explained in the methods section).	High<br>The authors show how interpretations relate to the data by providing excerpts of participants’ responses, and by naming percentage of participants endorsing various statements from the questionnaire.	Medium<br>Percentages of participants named. Peri- and post menopausal groups were compared using Chi square and p values are reported. Gives descriptive insight into use of the website but not its impact.	Self-reported accounts (may be biased or inaccurate); questionnaire assesses intention which is not the same as actual behaviour.
De Choudhury, Ringel Morris & White, 2014 LOG STUDY	High<br>The aim is clearly stated in form of three clearly and succinctly formulated research questions. The aim was to research the prevalence of health activities on different platforms, i.e. social media and search engines. The authors also aimed to characterize health activities on the different platforms and describe how people evaluate information obtained from these.	High<br>15 months of log data from a major Web search engine. The log data allows the examination of search behaviour in the ‘real World’.	High<br>It is described how relevant logs were identified. The study procedures and analyses of the data are described in detail.	Medium<br>The authors base their interpretations on both participants’ survey responses and log-based data. Some issues with interpretation of log data though, e.g. all instances of health mention on Twitter were considered information ‘sharing’, though some might be ‘seeking.	High<br>Descriptive analyses, Wilcoxon test. Yes these methods are appropriate for the aim of the study, i.e. analysing the prevalence of certain search behaviours, describing the characteristics of certain search activities.	Twitter and logs: assumes that certain queries/tweets relate to certain topics based on certain algorithms. Some queries may be wrongly classified or the algorithms may not assess what they assume to assess.

**Authors, date**	**Are the aims and objectives of the research clearly stated?**	**Is the research design clearly specified and appropriate for the aims and objectives of the research?**	**Do the researchers provide a clear account of the process by which their findings were reproduced? (methods)**	**Do the researchers display enough data to support their interpretations and conclusions?**	**Is the method of analysis appropriate and adequately explicated?**	**Main limitations**
De Choudhury, Ringel Morris & White, 2014 SURVEY	High<br>The aim is clearly stated in form of three clearly and succinctly formulated research questions (see above).	High<br>The survey allows the researchers to validate the information obtained from logs and get deeper insights. The survey sample was selected using census representative sampling in terms of age and gender.	High<br>The survey is described in detail and how it was coded and analysed.	Medium<br>The authors base their interpretations on both participants' survey responses and log-based data. Some issues with interpretation of log data though, e.g. all instances of health mention on Twitter were considered information 'sharing', though some might be 'seeking.	High<br>Open coding and descriptive analyses (percentages), Wilcoxon test to test for differences between ratings of search engines and Twitter.	Survey: Retrospective self-reported accounts – may be biased and/or inaccurate. All survey respondents were Twitter users so perhaps biased towards younger / tech savvy users.
Fiksdal et al., 2014	High<br>The aim is clearly stated in a clearly and succinctly formulated sentence (gain a deeper understanding of online health-searching behavior in order to inform future developments of personalizing information searching and content delivery)	High<br>Clearly specified (focus groups with 4-5 participants per group). This is appropriate for gaining a deeper understanding of a phenomenon.	Medium<br>Yes methods are described; sampling, the semi-structured moderator guide for the focus groups, and the analysis are described.	High<br>The authors provide quotes that illustrate the themes they have drawn out.	Medium<br>The method (grounded theory) is appropriate for qualitative data but it is not described in sufficient detail.	Retrospective self-reported accounts – may be biased and/or inaccurate. Qualitative study, therefore limited generalisability.
Fox & Duggan, 2013	Medium<br>The aim could have been described more clearly. While the report describes the Pew Internet & Americal Life Project and what it does, it would have been helpful had the authors described the aim in a clearly marked succinct sentence.	High<br>Cross-sectional population-based survey using telephone interviews. Appropriate approach for a nationwide, population-based study.	High<br>The methods are described clearly in the report.	High<br>The report summarises findings. The authors clearly describe trends in survey responses and any interpretations are based	High<br>Descriptive statistics. Appropriate for a population-based study aiming to describe prevalent behaviours/activities.	Survey: Retrospective self-reported accounts – may be biased and/or inaccurate.

Authors, date	Are the aims and objectives of the research clearly stated?	Is the research design clearly specified and appropriate for the aims and objectives of the research?	Do the researchers provide a clear account of the process by which their findings were reproduced? (methods)	Do the researchers display enough data to support their interpretations and conclusions?	Is the method of analysis appropriate and adequately explicated?	Main limitations
Hay et al., 2008	High<br>The aim is described succinctly in a clearly marked sentence. The aim was to understand the extent and reasons for online research prior to first appointments for newly diagnosed rheumatology patients.	High<br>Observational mixed methods study, using a survey and interviews pre and post appointments.	High<br>It is described what questions were asked of patients, when, and in what setting and how patients were identified.	High<br>The authors describe which tests were used to test which associations. They use descriptive statistics to describe the prevalence of online health seeking. They provide p values for associations they describe as significant. For categories from qualitative analyses, they provide quotes, and the percentage of the sample supporting these categories.	Medium<br>The authors use descriptive statistics to describe the sample and prevalence of different behaviours, and correlations, chi square tests and logistic regression to test for associations. These tests are appropriate. A power analysis could have been mentioned. The qualitative analysis seems appropriate but no specific methodology was mentioned – this could have been described in more detail.	Survey: Retrospective self-reported accounts – may be biased and/or inaccurate.
Keselman, Browne & Kaufman, 2008	High<br>The aim is described in two clear succinct sentences. The aim was to explore users' information seeking difficulties by conceptualizing information seeking as a form of hypothesis testing, and to examine the role of users competencies in online information seeking.	High<br>Qualitative interview and Think Aloud study. Appropriate for the aim of exploring phenomenon and for assessing Web use behaviour.	Medium<br>Methods are described and it is clear how participants were interviewed, which tasks they completed and how this was assessed (Think Aloud). Participants' ages are not mentioned!	High<br>The authors cluster participants into 3 clusters based on their search behaviour and then describe in detail the behaviour and experiences of participants in each cluster; they provide examples and quotes for each cluster and the number of participants in each cluster.	Medium<br>The authors use semantic analysis and thematic analysis which is suitable for qualitative data. The process of qualitative analysis (e.g. how were codes developed?) could have been described in more detail.	11/20 participants were employees of the US National Library of Medicine, all had some University education, 52% had a postgraduate degree), so biased sample. Qualitative analysis: provides some ideas but does not allow inferences to the general population. Laboratory setting: limited external validity.

Authors, date	Are the aims and objectives of the research clearly stated?	Is the research design clearly specified and appropriate for the aims and objectives of the research?	Do the researchers provide a clear account of the process by which their findings were reproduced? (methods)	Do the researchers display enough data to support their interpretations and conclusions?	Is the method of analysis appropriate and adequately explicated?	Main limitations
Lauckner & Hsieh, 2013	High<br>The aim is described clearly and succinctly in form of 4 hypotheses and 2 research questions. Does the position and frequency of serious conditions in search results affect perceived severity and susceptibity, and is this related to negative emotional outcomes? Do health literacy and experience with online health seeking moderate these relationships?	High<br>2x2 within-subjects design. Yes this is appropriate to test the research hypotheses, to test for interaction and moderation effects. The experimental design means it is appropriate to test for causal links which is part of the aims.	High<br>The experimental conditions are described in detail as well as the measures used to operationalise the variables of interest.	High<br>For each hypothesis and research question the authors describe the strength of associations (correlations), and significance values for main effects and interactions.	Medium<br>The authors use ANCOVA which is suitable to test for main effects and interactions, and path analysis to explore the causal chain of effects. A power calculation should have been mentioned.	Sample consists only of University undergraduates – biased.<br>Experimental setting: limited external validity.
Luger, 2014	High<br>The aim is described in a clear and succinct sentence. The aim was to explore older adults' online health seeking to determine the cognitive and diagnostic processes involved.	Medium<br>2x2 within-subjects, mixed methods design (embedded qualitative Think Aloud element). It is not clarified why they chose to vary the two factors (search tool and symptom vignette) and this is not incorporated into the analysis.	High<br>The conditions are described in detail, as well as recruitment strategies, eligibility criteria, and measures used to assess outcome measures.	High<br>The authors use qualitative analysis of Think Aloud to demonstrate participants' search behaviours. The authors report the percentage of participants engaging in different behaviours (p values and correlations are described for quantitative analysis but these results are not relevant for the review and are thus not reviewed here).	Medium<br>The Think Aloud method is appropriate to explore participants' search behaviour. The authors could have described their qualitative methodology and synthesis method in more detail.	All participants had university education, 52% postgrads – biased sample. Experimental setting: limited external validity
Medlock et al., 2015	High<br>The aim is described in a clear and succinct sentence. The aim was info sources seniors who use the Internet use for health information.	Medium<br>Online survey with convenience sampling via a Christian seniors organisation. The survey method is appropriate for the aims, but the sampling method may have resulted in bias.	Medium<br>Methods are described, however not eligibility criteria for participants are mentioned; perhaps an age cut-off would have been useful to focus on seniors?	High<br>The authors describe the sample based on responses to the questionnaire.	High<br>Linear regression was used to assess associations between variables. False discovery rate used to adjust p-values for multiple hypothesis testing (good methods – this is often not taken into account).	Survey: Retrospective self-reported accounts – may be biased and/or inaccurate.<br>Convenience sampling via a Christian senior's organisation.

**Authors, date**	**Are the aims and objectives of the research clearly stated?**	**Is the research design clearly specified and appropriate for the aims and objectives of the research?**	**Do the researchers provide a clear account of the process by which their findings were reproduced? (methods)**	**Do the researchers display enough data to support their interpretations and conclusions?**	**Is the method of analysis appropriate and adequately explicated?**	**Main limitations**
Morgan et al., 2014	High<br>The aim is described in a clear and succinct sentence: The aim was to describe what information people seek from a US website about genetic and rare diseases, and why, and compare this across two years.	High<br>Longitudinal, observational analysis of inquiries posted to a website. Suitable for the aim of describing website usage.	High<br>The website from which the inquiries were obtained is described in detail, as well as criteria used to choose which inquiries to include, the sample of inquiries (number etc.) and which data was available for users posting the inquiries.	High<br>The authors use descriptive statistics to describe the users, and name the percentages of inquiries relating to specific topics/questions. Inferential statistics are used to compare differences between inquiries of the two years (2006 and 2011)	High<br>The authors use content analysis to analyse the questions, which is appropriate for their aim of systematically describing the written content on the website.	Sample in 2006 (n = 68) is considerably smaller than in 2011 (n = 210).<br>Specific to genetic diseases.
Mueller et al., 2016	High<br>The aim is described succinctly; the aim was to assess the feasibility of testing an online lung cancer symptom appraisal tool in an online randomised trial	High<br>A pilot study replicating the design intended for a full randomised trial was conducted, which is suitable for assessing feasibility.	High<br>The study design, the intervention (the symptom appraisal tool) and the statistical methods used are described.	Medium<br>The researchers conduct inferential statistics, although data collection was still underway; the researchers acknowledge this limitation, however, and advise to interpret with caution.	High<br>Chi-square for categorical, Mann Whitney U test for non-normal data is appropriate. Bonferroni corrections to account for error accumulation. Effect sizes are reported.	Sample size of N=97 across 4 experimental conditions is quite small, and likely underpowered for inferential statistics.<br>Differential dropout limited the validity.
Norr, Capron & Schmidt, 2014	High<br>The aim was to investigate whether viewing medical websites may adversely affect anxiety sensitivity (AS), and whether this is moderated by intolerance of uncertainty.	Medium<br>Experimental design; participants were randomised to view either websites about symptoms or general health websites. Appropriate to determine causal effects. No power calculation mentioned. A convenience sample of undergraduate students is used so potential bias (younger, female, educated)	High<br>Participants, measures, procedures and experimental manipulations are described.	High<br>The authors clearly base their conclusions on the findings of the study, and are justified in drawing causal conclusions due to the experimental design.	High<br>The authors use chi square and t tests to test whether participants in the experimental and control conditions differed. Hierarchical regression to assess whether the experimental manipulation predicts AS.	Experimental setting: limited external validity.

Authors, date	Are the aims and objectives of the research clearly stated?	Is the research design clearly specified and appropriate for the aims and objectives of the research?	Do the researchers provide a clear account of the process by which their findings were reproduced? (methods)	Do the researchers display enough data to support their interpretations and conclusions?	Is the method of analysis appropriate and adequately explicated?	Main limitations
North et al., 2011	High<br>The aim is described in a succinct clear sentence: The aim was to establish what symptoms internet users tend to look up online, and whether this differs from telephone triage use.	High<br>Cross-sectional analysis of clicks on a health website and calls to a telephone triage system. Suitable for comparison.	High<br>It is described which data were included in analysis (clicks to specific symptoms on the Mayo symptom checker website, and calls to Ask Mayo Clinic) and how data were categorized for analysis.	High<br>The authors report correlations between calls and click relating to specific symptoms as well as Odds Ratios, which illustrates how use of a website differs from use of a telephone triage system. The authors report the strength of associations (correlations) and significance values to support their interpretations.	High<br>Odds Ratio are suitable to determine which symptoms are researched more on the Internet vs. telephone, and correlations are suitable to show whether there are relationships between use of internet and telephone for specific symptoms.	Conclusions about users' motivations were based on analysis of clicks rather than asking users directly. This may not reflect true motivation.
Perez et al., 2015	High<br>Described clearly. The aim was to describe Internet search processes, and identify demographic and personal characteristics associated with use of System 1 and System 2 processing (system 2: including a hypothesis testing and evidence gathering, system 1:all others)	High<br>Exploratory, mixed-methods study with experimental randomisation to one of two symptom vignettes and Think Aloud.	Medium<br>It is described how participants were recruited, eligibility criteria, study procedure, how data were collected and coded.<br>Problematic: "Unless the participant inquired, participants were not informed that the symptoms were suggestive of influenza or meningitis." Does this mean participants were told if they asked during the experiment?? That would reduce the validity of findings.	High<br>It is described in detail how search behaviour was coded/categorised into classifications, and how this data was inputted into a logistic regression model.	Medium<br>Logistic regression is appropriate to establish predictors of whether system 2 was used or not. However, a sample size of n = 78 seems small for this analysis, and no power calculation was reported.	Experimental setting: limited external validity.
Powell et al., 2011	High<br>The aims are described in a clear, succinct sentence. The aims was to identify the characteristics and motivations of online health information seekers accessing the NHS Direct website.	High<br>Survey with embedded qualitative interview study. Suitable to identify characteristics, and explore motivations.	Low<br>The interviews are described, though more detail could have been provided on the analysis. The survey measure is only described very vaguely, no details provided.	High<br>For the survey, the authors provide descriptive data (proportions) and measures of the strength of associations. For the interview data, the authors illustrate their themes using participant quotes, with demographic information on the participant.	Medium<br>Descriptive statistics and inferential statistics for the survey are suitable. Difficult to assess suitability of the qualitative analysis as few details are provided.	Retrospective self-reported accounts – may be biased and/or inaccurate. Only focused on NHS Direct users – not generalizable to other users.

Authors, date	Are the aims and objectives of the research clearly stated?	Is the research design clearly specified and appropriate for the aims and objectives of the research?	Do the researchers provide a clear account of the process by which their findings were reproduced? (methods)	Do the researchers display enough data to support their interpretations and conclusions?	Is the method of analysis appropriate and adequately explicated?	Main limitations
Powley et al., 2016	High<br>The aims are described in a clear, succinct sentence. The aims was to assess whether patients with inflammatory arthritis use the Internet to appraise their symptoms, and to assess outcomes of symptom checker tools when used by patients.	High<br>A survey and observational study (observation of patients using symptom checker tools) is suitable to evaluate Internet use and outcomes of symptom checker tools.	Medium<br>Mostly clear, but it is not stated whether patients completed the study before or after obtaining their diagnosis. This might affect how they complete the symptom checker tool. It is not clearly stated how patients' searches and outcomes were recorded (video?audio? screenshots?)	High<br>The authors show the number and proportion of patients reporting Internet use, which information patients inputted into the symptom checker tools, and which advice and diagnoses are suggested by the tools.	Low<br>The authors mostly just report counts and proportions. But the authors use some inferential statistics though their sample size is very small (N = 34) and likely underpowered; no power analysis is mentioned.	Small sample size; likely underpowered for inferential statistics. Retrospective self-reported accounts – may be biased and/or inaccurate. For the observational part (observing patients completing a symptom checker tool), validity is questionable – Would users in real settings rely on just symptom checkers?
Rice, 2006	High<br>The aims are described in a clear, succinct sentence. The aim was to understand what influences online health seeking, what the reported benefits of online health seeking are, and to identify similarities among online activities.	Medium<br>Cross-sectional survey design; analysis of existing dataset from Pew Internet and American Life project. This is suitable for description but not to identify influencing factors.	Medium<br>The datasets are described as well as which variables were included. Methods and results appear to be mixed together so it is difficult to ascertain analysis methods used.	High<br>The authors draw conclusions about which variables are associated with online health seeking and provide significance values as well as R square and standardised beta coefficients for their models.	High<br>The authors use cross-tabulations, logistic regressions, and multidimensional scaling. These methods are appropriate to establish predictors of online health seeking and to identify similarities between Internet activities.	Limited relevance as they do not distinguish between those who search pre and post diagnosis.<br><br>Retrospective self-reported accounts – may be biased and/or inaccurate. Only asked about using the Internet to self-diagnose without seeing a doctor, which may exclude those who researched online and then saw a doctor.
Teriaky, Tangri & Chande, 2015	High<br>The purpose of this study is to understand how outpatients awaiting initial gastroenterology consultation seek medical information on the Internet.	Medium<br>Cross-sectional survey with patients awaiting consultation at a gastroenterology clinic. Appropriate to describe how individuals used the internet. Sample size seems small (N = 87) and no power calculation reported.	High<br>Design, procedure, material and methods are described in sufficient detail.	High<br>Conclusions are based on the associations reported in the results.	High<br>The authors use descriptive statistics, and chi square / t tests to test differences between those who used the Internet and those who did not.	Retrospective self-reported accounts – may be biased and/or inaccurate.

Authors, date	Are the aims and objectives of the research clearly stated?	Is the research design clearly specified and appropriate for the aims and objectives of the research?	Do the researchers provide a clear account of the process by which their findings were reproduced? (methods)	Do the researchers display enough data to support their interpretations and conclusions?	Is the method of analysis appropriate and adequately explicated?	Main limitations
Thomson, Siminoff & Longo, 2012	High<br>The aims is described in a clear succinct sentence: The aim was to explore characteristics of colorectal cancer patients who used the web to appraise symptoms prior to diagnosis.	High<br>Cross sectional survey design. This design is appropriate for the aim of establishing characteristics of those using the Web for symptom appraisal and describing their experiences.	High<br>It is described how patients were identified, eligibility criteria, and which questions were asked during interviews and how these were conducted.	Medium<br>The authors provide correlations and significance values for the factors they report as associated with Web use. However they conclude WUPD did not 'influence' patient delay. This study design does not allow causality inferences.	High<br>It is described how interview data was coded to allow quantitative analysis. The authors use t-tests, chi-square tests, ANOVA, logistic & linear regression, and correlation analysis. These methods are appropriate to determine the characteristics of those who use the Web. The sample size seems sufficiently large.	Retrospective self-reported accounts – may be biased and/or inaccurate.
White & Horvitz, 2009 a<br>LOG STUDY	High<br>The aims are described in form of 2 clear research objectives: 1) to describe escalations that occur when users search for common symptoms and this escalates to queries about serious conditions, and 2) how this persists over several sessions.	Medium<br>Log-based longitudinal study (using logs from windows live toolbar). Log data can provide information about behaviours of 'real' users (but limited as it is based on assumptions).	Medium<br>It is described in detail how log data was obtained, how it was analysed to identify users who were searching for symptoms, and how their subsequent behaviour (escalation). But some numbers are only vaguely reported, e.g. the number of users from whom log data was collected ('hundreds of thousands').	Low<br>The authors describe how they identify escalations and interruptions and how this can interrupt subsequent searches. But the authors tend to overstate, concluding causal relationships from cross-sectional observational data and equating 'escalations' with increased anxiety. The validity of interruptions was tested by visual inspection by one of the authors – but even visual inspection cannot verify searchers' true intentions. The validity of other assumptions was not tested.	High<br>The authors provide descriptive statistics to describe user behaviour (percentages of logs). Tukey's *post-hoc* tests, independent measures t test, ANOVA, multiple regression analysis. These tests are appropriate to describe whether searches containing escalations differ from those without escalations.	Log based study – assumes that certain queries relate to certain topics based on certain algorithms. Some queries may therefore be wrongly classified or the algorithms may not assess what they assume to assess.
White & Horvitz, 2009 a<br>SURVEY	High<br>The aims are described in form of 2 clear research objectives: 1) to describe escalations that occur when users search for common symptoms and this escalates to queries about serious conditions, and 2) how this persists over several sessions.	Low<br>Cross-sectional survey. Survey is used to supplement and validate log data. This is appropriate but the log sample and survey sample are likely significantly different, as the survey sample is heavily biased (Microsoft employees).	High<br>The survey is described, i.e. which questions are asked and how participants were recruited.	Low<br>The authors describe the survey results. But the authors tend to overstate, concluding causal relationships from cross-sectional observational data. Furthermore they identified hypochondria through two simple self-reported items; a validated scale would have been more appropriate.	High<br>The authors provide descriptive statistics for the survey data.	The survey sample is heavily biased towards young, educated male respondents. Retrospective self-reported accounts – may be biased and/or inaccurate.

**Authors, date**	**Are the aims and objectives of the research clearly stated?**	**Is the research design clearly specified and appropriate for the aims and objectives of the research?**	**Do the researchers provide a clear account of the process by which their findings were reproduced? (methods)**	**Do the researchers display enough data to support their interpretations and conclusions?**	**Is the method of analysis appropriate and adequately explicated?**	**Main limitations**
White & Horvitz, 2009 b	High<br>The aims are described in a clear and succinct sentence: The aim was to explore how lay individuals use the Web to find explanations for symptoms, what activities they pursue and what their experiences are.	Low<br>Cross-sectional survey study. This is appropriate for exploring and describing behaviour and experiences of individuals. But the sample is heavily biased (Microsoft employees).	High<br>The survey described in detail, i.e. which questions were asked, how it was administered, how participants were identified and recruited.	Medium<br>The authors provide percentages when describing responses, and significance values when describing differences between respondent groups. Survey results are partly overstated, i.e. causal conclusions based on observational, cross-sectional data (the Web influences anxiety levels)	High<br>The authors use chi square test and independent t tests to test for significance of observed differences; appropriate test to determine differences between Web users, e.g. between those who report themselves as hypochondriacs compared to the rest.	Retrospective self-reported accounts – may be biased and/or inaccurate. The survey sample is heavily biased towards young, educated male respondents.
White & Horvitz, 2010 a	High<br>The aim is described in a clear and succinct sentence: The aim was to predict escalations in searches based on characteristics of websites visited.	High<br>Cross sectional log study using logs from windows live toolbar. This is an appropriate design for analysing and describing escalations based on web site characteristics.	Medium<br>The authors describe in detail how they identified and defined escalations, and which traits of websites they examined. But the exact number of logs mined for the study is not given.	High<br>The authors demonstrate the predictive value of their model and which website traits are significantly related to escalations.	Medium<br>The authors use logistic regression to establish a model predicting escalations based on website traits, however beta coefficients and R square values are not reported.	Log based study – assumes that certain queries relate to certain topics based on certain algorithms. Some queries may therefore be wrongly classified or the algorithms may not assess what they assume to assess.
White & Horvitz, 2010 b	High<br>The aim is described in a clear succinct sentence: The aim was to establish predictors of when searches for common symptoms lead to health care utilization.	Medium<br>Longitudinal sectional log based study. This is appropriate to analyse online user behaviour but less appropriate to determine behaviour the Web.	High<br>The authors describe which data was available, which logs were included/excluded, how they identified logs relating to common symptoms, and how they defined logs showing healthcare utilization intent.	Medium<br>The authors draw conclusions about website and user characteristics that are related to healthcare utilization intent, and demonstrate this using logistic regression analysis. But the measure of healthcare utilization intent may be flawed; it is not clear it searching for healthcare institutions reflects healthcare utilization intent.	Medium<br>The authors use logistic regression to predict healthcare utilization intent queries based on traits of web pages viewed as well as user characterisitcs. Beta coefficients were not reported.	Log based study – assumes that certain queries relate to certain topics based on certain algorithms. Some queries may therefore be wrongly classified or the algorithms may not assess what they assume to assess. Specifically: querying for healthcare facilities may not mean intent to visit them, and searching for symptoms may not mean self-diagnosis.

**Authors, date**	**Are the aims and objectives of the research clearly stated?**	**Is the research design clearly specified and appropriate for the aims and objectives of the research?**	**Do the researchers provide a clear account of the process by which their findings were reproduced? (methods)**	**Do the researchers display enough data to support their interpretations and conclusions?**	**Is the method of analysis appropriate and adequately explicated?**	**Main limitations**
White & Horvitz, 2012	High<br>The aim is described in a clear sentence. The aim was to explore how users search for medical concerns and particularly how these concerns impact on future behaviour, e.g. how this influences focus and attention of future searches.	High<br>Longitudinal log-based study. This is appropriate to study user behaviour over time.	Medium<br>The authors describe how they identified logs related to medical concerns and how they labeled these, and also define clearly the different transitions types examined (e.g. transition from symptom to serious illnesses, or symptoms to benign explanations). Exact number of users/logs not stated.	Medium<br>The authors back up their claims about user behaviour by describing the logs which they previously assigned to categories. However the authors make assumptions that are potentially flawed – e.g. that the user is searching pre-diagnosis, when they may be researching a diagnosed condition.	High<br>Descriptive statistics are used to describe user behaviour (e.g. percentage of logs symptoms related), and logistic regression was used to predict whether a web page was medical or not.	Log based study – assumes that certain queries relate to certain topics based on certain algorithms. Some queries may therefore be wrongly classified or the algorithms may not assess what they assume to assess.
White & Horvitz, 2013	High<br>The aims are described as 2 clear, succinct research questions: 1) whether snippets in search results are biased towards serious conditions when symptoms are entered into search engines and 2) how these snippets influence user behaviour.	High<br>Longitudinal log-based study. This method is suitable to examine result pages of search engines and user behaviour (hovering and clicks).	High<br>It is described in detail how log data was obtained, how it was analysed to identify users who were searching for symptoms, and how their subsequent behaviour (hovering over captions, clicks) was established.	High<br>The authors provide descriptive statistics (percentages) to describe results returned by search engines, and significance values to establish whether behaviours of users differ depending on whether snippets relate to serious or benign causes of symptoms.	Medium<br>The authors use t tests, ANOVA and Tukey-Kramer tests; these are appropriate to establish whether user behaviours differ depending on snippet content. The authors describe in detail how they developed their click prediction model based on the Dynamic Bayesian Network model. Because methods are described alongside results rather than all under a specific heading (‘statistical analyses) it is difficult to establish exactly which tests the authors used.	Log based study – assumes that certain queries relate to certain topics based on certain algorithms. Some queries may therefore be wrongly classified or the algorithms may not assess what they assume to assess.<br><br>Internal validity
Ybarra & Suman, 2006	High<br>The aim is described in a clear succinct sentence: The aim was to examine which user characteristics predict whether a Web user is likely to contact a health professional.	High<br>A national, cross-sectional, randomly sampled telephone survey. This method is appropriate to establish characteristics of individuals engaging in certain behaviour.	High<br>The authors describe how the survey was conducted, how participants were contacted and recruited, and which questions were asked.	High<br>The authors back up their claims by reporting significance values and odds ratios, percentages of individuals endorsing certain behaviours/characteristics. They do not draw causal conclusions.	High<br>Chi square, t-tests, ANOVA. These tests are appropriate to identify differences between those reporting contact with health professionals and those who did not.	Retrospective self-reported accounts – may be biased and/or inaccurate. The authors do not consistently distinguish between people seeking health information online to diagnose symptoms, and those seeking health information to look up an already received diagnosis.
