# Supplementary figures and images for: Web Use for Symptom Appraisal of Physical Health Conditions: A Systematic Review
Source: J Med Internet Res. 2017 Jun 13;19(6):e202. doi: 10.2196/jmir.6755 (PMC5487739; doi:10.2196/jmir.6755)

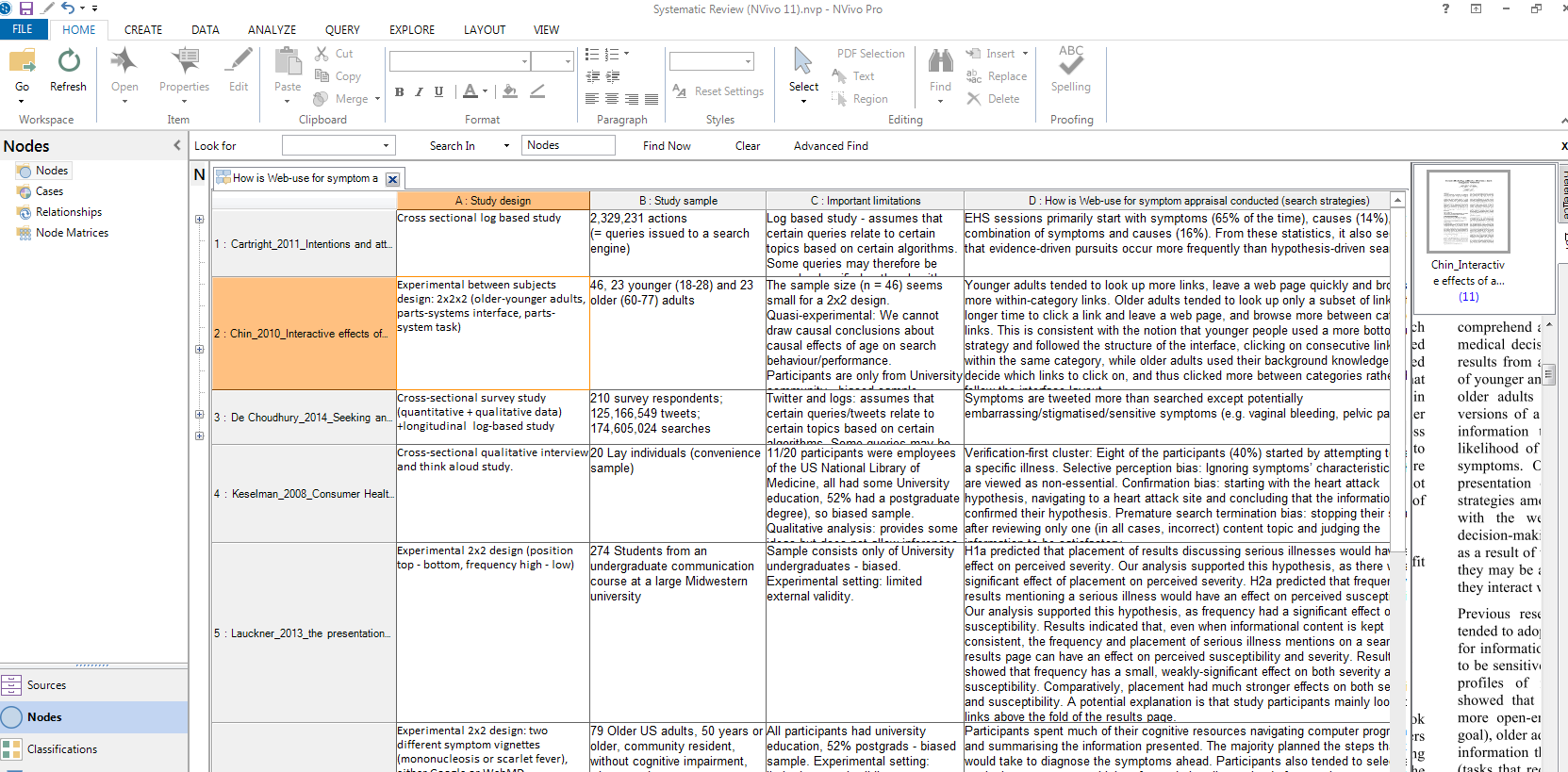

Supplement: Multimedia Appendix 4 [file jmir_v19i6e202_app4.png]
